# Multi-Elemental Analysis of Wine Samples in Relation to Their Type, Origin, and Grape Variety

**DOI:** 10.3390/molecules26010214

**Published:** 2021-01-04

**Authors:** Magdalena Gajek, Aleksandra Pawlaczyk, Malgorzata I. Szynkowska-Jozwik

**Affiliations:** Faculty of Chemistry, Institute of General and Ecological Chemistry, Lodz University of Technology, Zeromskiego 116, 90-924 Lodz, Poland; aleksandra.pawlaczyk@p.lodz.pl (A.P.); malgorzata.szynkowska@p.lodz.pl (M.I.S.-J.)

**Keywords:** wine samples, beverages, multi-elemental analysis, trace elements, ICP-MS, ICP-OES, CVAAS, PCA

## Abstract

Wine is one of the most popular alcoholic beverages. Therefore, the control of the elemental composition is necessary throughout the entire production process from the grapes to the final product. The content of some elements in wine is very important from the organoleptic and nutritional points of view. Nowadays, wine studies have also been undertaken in order to perform wine categorization and/or to verify the authenticity of products. The main objective of this research was to evaluate the influence of the chosen factors (type of wine, producer, origin) on the levels of 28 elements in 180 wine samples. The concentration of studied elements was determined by ICP-MS (Ag, B, Ba, Be, Bi, Cd, Co, Cr, Cu, Li, Mn, Mo, Ni, Pb, Rb, Sb, Sn, Sr, Te, Tl, U, Zn), ICP-OES (Ca, Fe, K, Mg, Ti), and CVAAS (Hg) techniques in 79 red, 75 white, and 26 rose wine samples. In general, red wines contained higher values of mean and median of B, Ba, Cr, Cu, Mn, Sr and Zn in contrast to other wine types (white and rose). In white wines (when compared to red and rose wines) higher levels of elements such as Ag, Be, Bi, Cd, Co, Li, K and Ti were determined. In contrast, rose wines were characterized by a higher concentration of Fe and U. The study also revealed that in the case of 18 samples, the maximum levels of some metals (Cd—8 samples, Pb—9 samples, Cu—1 sample) were slightly exceeded according to the OIV standards, while for Zn and Ti in any wine sample the measured concentrations of these metals were above the permissible levels. Thus, it can be stated that the studied wines contained, in general, lower levels of heavy metals, suggesting that they should have no effect on the safety of consumption. The results also showed higher pH level for red wines as a consequence of the second fermentation process which is typically carried out for this type of wine (malolactic fermentation). The highest median value of pH was reported for Merlot-based wines, while the lowest was for Riesling. It is assumed that dry Riesling has a higher content of tartaric and malic acid than dry Chardonnay grown in the same climate. From all of the studied countries, wines from Poland seemed to present one of the most characteristic elemental fingerprints since for many elements relatively low levels were recorded. Moreover, this study revealed that also wine samples from USA and Australia can be potentially discriminated from the rest of studied wines. For USA the most characteristic metal for positive identification of the country of origin seems to be uranium, whereases for Australia – strontium and manganese. Based on the highly reduced set of samples, it was not possible to differentiate the studied wine products according to the grape variety other than Syrah, and partially Chardonnay. Since all the Syrah-based samples originated from the same country (Australia) thus, the observed grouping should be more related with the country of origin than the grape variety.

## 1. Introduction

Wine is one of the oldest known alcoholic beverages, which is commercially and domestically produced as a result of grape must fermentation. Wine samples are recognized as a relatively complex matrix from an analytical procedure perspective, since besides water, they also contain ethanol (typically between 9 and 15%), organic acids (such as malic acid, tartaric acid, citric acid, acetic acid or lactic acid—as a result of a malolactic fermentation), and carbohydrates (e.g., polycyclic aromatic hydrocarbons) [1].

In recent years, many studies have been conducted in which the content of trace elements and stable isotope ratios as indicators of the origin of the food industry products have been discussed [2,3,4,5,6]. Wine analysis seems to draw much attention because of its importance in assessing the quality of a food product, possible verification of adulteration, and analysis of its position in the production chain in the agriculture and the food industries.

In addition to analyzing organic ingredients such as acids, sugars, flavonoids, and aromatic and flavoring compounds, analysis of the elemental composition is an important parameter of quality assessment. It has been proven that apart from the main wine components, some compounds that are present at relatively low levels can significantly affect the wine characteristics in terms of wine quality by changing the taste or color. Therefore, the control of the content of individual substances is necessary across the entire production process from the grapes to the final product from the organoleptic and nutritional points of view. 

The level of heavy metals in wine may originate from many different sources. Metals migrate from rocks to soil and then from the soil to fruit through the plant’s roots. As a consequence, the levels of some trace elements will be directly related with the soil composition and properties as well as with the plant uptake processes. Thus, the elementary composition of wine should illustrate the geochemistry of the soil of the vineyards in which the fruit was grown, and the condition of the plant from which the drink was produced. There are studies that have shown that wines from grapes from the same vineyard tend to be most similar, regardless of the time of their storage or grape variety. This theory clearly states that ‘fingerprints’ of wine are relict soil signatures that survive metabolic and winery processing. The uptake of elements is primarily influenced by pH or complexing agents [7]. However, several other factors such as environmental pollution, agricultural practices (e.g., treatment by fertilizers, pesticides, and fungicides), climate change, grape variety, local conditions, or vinification processes (wine production, wine processing as filtration and clarification, conservation, and bottling, especially when during the wine making process, the grape material stays in contact with various substances for a long period of time like stainless steel, wood from a barrel, or glass from the bottle) can significantly change the natural elemental composition of wine and affect the relationship between its composition and the content of elements in the soil. It should be noted that metals play a significant role in the efficient alcoholic fermentation of wine and influence their organoleptic characteristics (flavor, freshness, aroma, color, and taste), mostly by precipitation and clouding processes [8,9,10]. Below few examples are given of studies in which aspects regarding the relationship between metals content and wine parameters were discussed. It was revealed that, for instance, the content of K, Ca, and Na can be easily changed by additives used in the production process (e.g., tartaric acid) or by cooling the wine dishes because these elements can easily interact with tartaric acid and create hydrogen tartrate. Formed molecules can easily precipitate as a consequence of temperature changes [11]. In turn, bentonites used for wine clarification can contaminate wine with sodium and aluminum ions and contribute to an increase in the concentration of rare earth metals. For this reason, the clarification of red wines, especially young ones, is prohibited in France [12]. It has been reported that the concentration of several elements (Cd, Cr, Pb, Al, Cr, Fe, Ni, Pb, and V) increases as a result of contamination from wine-making equipment made of stainless steel, bronze, or brass [13]. Some elements such as Ni, Cr, Li, Cd, Co, Mn, Fe, or Ti may migrate to the drink from the glass bottles during the maturation and aging of wine in bottles because these elements are added during the production of glass as color additives [14,15]. Moreover, the metals present in wine are the result of not only their sorption from the soil or the production and storage methods, but may also originate from the deposition of some metals in the environment. For example, the increased Pb content in wine may be related to the location of vineyards near high density traffic roads since some lead traces from fuels are emitted into the environment from the combustion process. On the other hand, in vineyards located near sea areas, increased sodium content can be identified. Some elements that have a detrimental effect on the stability of wine are closely monitored during the wine process production [16]. It is necessary to mention Cu, Fe, Zn, or Al, which can act as catalysts and cause the oxidation of wine components, which in turn can lead to the formation of haze or loss of valuable flavor. All presented examples undoubtedly show that in many cases, the possible discrimination of wine samples seems to be quite complicated, if not impossible since many factors can effectively change the natural fingerprint of these products. However, as suggested in some papers, suitable discriminant analysis can accurately classify the wines (e.g., according to vineyard) [7].

In order to protect consumer health against toxic substances and ensure the proper quality, there is a strong need to control the food and beverage industry from the origin to the final product. This goal is achieved by introducing limits of maximum acceptable concentrations of hazardous substances. For instance, the intergovernmental organization of a scientific and technical nature, the International Organization of Vine and Wine, has established the maximum admissible levels of some metals (e.g., Al, As, Cd, Cu, Pb, Ti, Zn) (Table 1). This organization is made up of 47 member states (including the three biggest international wine producers, namely, Italy, France, and Spain) with the European Union as a party, which granted a particular status. Moreover, some countries have set their own maximum permissible levels of elements and substances that cannot be exceeded in alcoholic beverages.

A brief and concise literature review included in Table S1 (Supplementary Materials) shows some selected papers in which the content of chosen metals in wines was determined. As can be seen, the most popular techniques applied for elemental composition studies are the ones based on ICP as a source of excitation and ionization [5,6,21,22,23,24] and on atomic absorption phenomena [2,3,19,25,26,27,28]. Authors have mostly analyzed regional products [2,3,5,19,22,24,25,26,27,28,29,30,31], and to a lesser extent, have focused on wine products outside the country of origin [6,21,26]. The majority of papers focused on red wine analysis [5,6,19,21,22,23,24,26,27,28,29,31], while white wine studies were the next most popular [2,5,19,22,23,24,26,27,29], however, papers on rose wines are quite rare [24,27]. The parameter mostly verified against the elemental composition of wine is the country of origin [3,5,21,22,26,28]. The positive discrimination of wines seems to be more successful when the samples from different regions, but within the same country, were studied [3,5,22,28]. In addition, the influence of factors such as the brand [19,23,27,31], grape variety [6,23], or the wine type [6,24] on the concentration of chosen elements have also been discussed. Based on the literature review, it can be stated that many authors have, for classification/discrimination purposes, determined not more than 13 elements [2,3,6,19,23,25,27,28,30,31]. The analysis of 30 elements or more in wine samples is relatively uncommon [5,21,22,24]. What should be underlined is the fact that the obtained concentrations for some heavy metals can vary greatly. As indicated in the literature, about 50 substances are typically determined in wine samples including eight elements identified as ash constituents, 25 trace elements, and about 20 ultra-trace elements and additionally radioactive elements as well as rare earth elements. In Table 2, the levels of the mentioned components were divided into ranges in which they can be commonly found in commercially available wines [8]. According to some sources, detailed elemental analysis, supported by chemometric evaluation, is an excellent tool for determining the region and origin of wines [32].

The main objective of this research was to evaluate the influence of chosen factors (type of wine, grape variety, origin–geographical indication) on the levels of 28 elements (Ag, B, Ba, Be, Bi, Ca, Cd, Co, Cr, Cu, Hg, Fe, K, Li, Mg, Mn, Mo, Ni, Pb, Rb, Sb, Sn, Sr, Te, Ti, Tl, U, Zn) in 180 wine samples. In this paper, the results gathered for red (79), white (75), and rose (26) wines originating from 23 countries are presented with the aim of finding and identifying existing correlations between the measured elemental content by inductively coupled plasma-mass spectrometry (ICP-MS), inductively coupled plasma-optical emission spectrometer (ICP-OES) and cold vapor-atomic absorption (CVAAS) techniques and wine characterization parameters. The gathered values for selected elements were tested against the maximum admissible levels at the international level (OIV standards). Due to the huge and complex dataset that was obtained in this study, some basic statistical tests and unsupervised classification methods were employed to present statistically significant differences among the studied groups to indicate whether there are any patterns or trends, to explore possible wine and variable classes, or to reduce the number of variables in order to explain the data variation as much as possible. The potential of the used instrumental techniques (ICP-MS, ICP-OES, and CVAAS) for possible wine sample categorization and discrimination is also discussed. The presented outcomes can broaden the knowledge in the field of wine fingerprinting even though many papers can be found in the literature in which wine classification based on their elemental composition have been undertaken. However, to our knowledge, there are a limited number of studies in which such a huge variety of samples was collected, representing at the same time different geographical regions (23 countries, only 42 out of 180 samples originated from Poland), types (red, white, and rose), and grape varieties (separate analysis was performed for nine grape varieties corresponding to the 38 wine samples, for which only one grape variety was declared by the producers). Thus, in this study, not only the results for a significant number of rose wines are presented (which is quite uncommon in the literature), but many parameters are also discussed against which the obtained results were tested.

## 2. Materials and Methods

### 2.1. Samples

A total of 180 wine samples (79 red, 75 white, and 26 rose originating from different countries (23) were chosen for the elemental analysis by the ICP-MS, ICP-OES, and CVAAS techniques. Alcohol samples selected in this study consisted mostly of brands of wines widely available on the Polish market, which can be easily found in wine warehouses and supermarkets. The studied samples varied both in terms of their type (red, white, rose, sweet, semi-sweet, semi-dry, dry), price, and origin. All samples produced outside Poland (138 samples mostly from Italy, France, and USA) came from unsuspicious origins and were produced from the traditional varieties. Among the samples from Poland, a few wines were home-made (two out of 42 samples). Samples were collected directly from the shops, and after the original bottles were opened, they were transferred into special plastic containers and stored at 3–4 °C until analysis.

All important information about the origin, type of wine as well as the price of the sample is given in Table S25 (Supplementary Materials). The wine names were coded and the manufacturer’s names of the wines are not given in this paper. Basic characteristics of the tested samples in relation to their origin and grape variety are included in Table 3.

### 2.2. Sample Preparation and Equipment

#### 2.2.1. ICP-ToF-MS and ICP-OES

**Figure 1:**
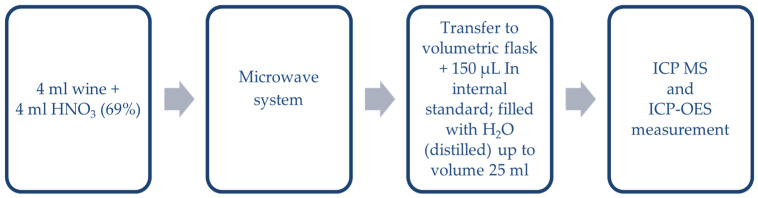
Procedure of sample preparation.

ICP techniques require a special preparation step before the analysis, which includes sample mineralization (Ultrawave system, Milestone, Via Fatebenefratelli, Italy) according to the procedure shown in Figure 1. In the literature review presented in Table S1 (Supplementary Materials), it can be concluded that the samples were only directly diluted [3,5,22,23,24,27], mineralized in HNO_3_ [2,6,21,23,26,28,31], or mineralized in both HNO_3_ and H_2_O_2_ [19,22,25,30]. The procedure finally adopted in this work was used previously by authors for the preparation of whisky samples [4].

The ICP-MS analytical technique (OptiMass 8000, by GBC, Melbourne, Australia) was applied to determine and quantify most of the metals in wine samples based on the chosen isotopes (^107^Ag, ^11^B, ^138^Ba, ^9^Be, ^209^Bi, ^111^Cd, ^59^Co, ^52^Cr, ^63^Cu, ^7^Li, ^55^Mn, ^95^Mo, ^60^Ni, ^208^Pb, ^85^Rb, ^121^Sb, ^118^Sn, ^88^Sr, ^125^Te, ^48^Ti, ^203^Tl, ^238^U, ^66^Zn). This instrument was equipped with a time-of-flight analyzer, which guarantees almost simultaneous types of measurements for the whole mass spectra. Concentrations of Ca, K, Mg, Fe, and Ti were determined by the ICP-OES (Thermo Scientific, ICAP 7000 series, Waltham, MA, USA). The ionic lines of Ca (393.366 nm), Mg (279.553 nm), K (766.490 nm), Fe (259.92 nm), and Ti (334.941 nm) were measured. The concentration of Ca, Mg, and K was determined in the radial plasma position, while the levels of Fe and Ti were assessed in an axial plasma setting.

Three replicates were performed for each wine sample for both techniques and in the case of the ICP-ToF-MS technique, three spectra were collected with the acquisition time of 3 s. In terms of the ICP-OES instrument, the exposure time for the UV range was 15 s, while for VIS it was 5 s. The possible drift of signal was monitored based on the intensity of signal for In used as an internal standard. Information on the operating conditions for the multi-elemental analysis of wines on the ICP-OES and ICP-ToF-MS spectrometers are presented in Table 4.

Before measurement by the ICP-MS and ICP-OES techniques, it was necessary to optimize the parameters of both spectrometers. This process was conducted based on the measurement of the “Tuning Solutions” with a known composition and concentration of the studied metals. During the optimization process, attention was paid in particular to the following parameters: the torch position, plasma power, gas flows, and the detector.

For multi-element measurement, it was necessary to prepare calibration curves based on a standard solution of Merck VI (Multi-element ICP standard, Merck, Darmstadt, Germany) with an initial concentration of 10 ppm elements contained in it and some single element standards of In (ICP class, Merck, Darmstadt, Germany), Sb (ICP class, Merck, Darmstadt, Germany), Sn (ICP class, Chem Lab NV, Zedelgem, Belgium), and Ti (ICP class, Radian International LLC, Austin, TX, USA) with an initial concentration of 1000 ppm, respectively. The calibration standards were mixed in such a way that the same matrix was present in the standards (e.g., separate calibration curve was performed for Sn and Sb standards, being in the matrix of HCl, which were finally mixed together). Preparation of standards was carried out by the subsequent dilution method. Measurements for wine and standard solution samples were recorded during a two-day analytical cycle. The calibration checks were repeated after every 20 analyzed samples. The blank samples was prepared in the same way as the studied samples (4 mL of concentrated nitric acid was put into the microwave system, transferred into 25 flask and diluted into the final volume with water). The correctness of the applied procedure was verified based on the analysis of the certified reference material of TMDA 64 (fortified lake water sample by National Water Research Institute, Burlington, Halton, ON, Canada).

#### 2.2.2. Mercury Analyzer

In this study, an automatic mercury analyzer MA-3000 (Nippon Instruments Corporation, Tokyo, Japan) was used to determine the total mercury content in the wine samples. The analytical procedure was analogous to the one described by Pawlaczyk et al. (2019) [4]. The limit of quantification was assessed as the sum of the mean results for 10 measurements for empty ceramic boats and six times the standard deviation and was accepted as approx. 0.23 ng of Hg.

#### 2.2.3. pH Meter

A Basic 20+ pH-Meter (CRISON INSTRUMENTS S.A., Barcelona, Spain) was used to measure the pH of the tested wines. The device was equipped with a magnetic stirrer and automatic temperature stabilization. The measurement was made using a combined electrode consisting of a glass electrode and a silver chloride electrode placed in one holder. Prior to the pH measurement, the device was calibrated using technical buffers at pH 4.01, 7.00, and 9.21 (HACH Company, Düsseldorf, Germany), respectively. Measurements of the pH of wine samples were carried out during a one-day analytical cycle. For all samples, three repetitions were made. The final results are expressed as a mean of individual measurements. The calibration was repeated after every 20 analyzed samples.

### 2.3. Data Analysis

STATISTICA 10 (New York, NY, USA) software was used for statistical and multivariate analysis. In order to use the appropriate statistical tests for analysis, it was first necessary to specify the type of distribution of the analyzed variables. For this purpose, Kołmogorov–Smirnov test was used to assess the distribution of all analyzed samples for the accepted level of significance α = 0.05. In the case when the null hypothesis regarding the normal distribution was rejected, a nonparametric test—Kruskal–Wallis test—was used to analyze the data. This test is typically applied to study the potential statistically important differences in the concentration of chosen variables among various group pairs.

All the gathered results were also presented in the form of box and whisker plots. This type of graph well illustrates the differences in the concentration of various variables within the studied objects in reference to the selected criteria.

Additionally, principal component analysis (PCA) and hierarchical cluster analysis (HCA) were performed. Column auto scaling was made before the implementation of CA analysis. Cluster analysis was chosen with Ward’s method and the squared Euclidean distance was applied to calculate the cluster distance.

## 3. Results and Discussion

### 3.1. Level of Metals in Analyzed Wine

Nowadays, there is an increasing public awareness about the harmfulness of chemical substances present in food. For this reason, there is a strong need to carry out scientific research in this field that can broaden our knowledge of the importance of dangerous ingredients in products consumed in our diet, which may potentially have a negative impact on our health. Wine samples are quite frequently studied mostly in terms of the content of heavy metals (such as Pb, Cd, Ni, Hg) and organic and inorganic substances (such as polyphenols or fertilizer residues). Besides the safety of the consumption of wine, this type of material can also be used in other approaches. According to some authors, the origin of samples recognized as the so-called history of product can be elucidated by determining the concentration of trace elements. This type of analysis is regularly performed in archeology, geology, forensic science, and most recently, in biological sciences. The wine matrix seems to be particularly suitable due to quite homogenous matter and the possibility of collecting a considerable number of representatives within a relatively large areas. For this purpose, the term enologic taxonomy is typically applied, which consists of a two-step approach. In the first step, as many elements as possible are determined by multi-element methods of analysis, which enables the determination of a large number of elements in a simultaneous way with sufficient reproducibility and sensitivity, like that in our case. In the second stage of the enologic taxonomy, the gathered data are assessed and characterized by proper mathematic methods and models. This approach can be potentially used to theoretically identify different wine categories created based on pattern recognition, according to location (geographic origin), vine variety, vintage year, etc. [8]. This type of wine characterization was employed in our case and an attempt was made to differentiate the studied samples.

In this study, the level of 28 elements was determined in 180 samples of wine. The concentration of Ag, B, Ba, Be, Bi, Cd, Co, Cr, Cu, Li, Mn, Mo, Ni, Pb, Rb, Sb, Sn, Sr, Te, Tl, U, and Zn was determined by the ICP-MS technique, while the level of Ca, Fe, K, Mg, and Ti was measured by the ICP-OES technique. The total content of Hg was assessed by the CVAAS technique.

Most of the selected metals were analyzed in all wine samples with some exceptions. For some samples, the obtained results were below the set detection limits. Thus, Li, U, and Ni were not determined in one sample; Cr and Zn were not determined in two samples; Be and Pb were not determined in three samples; B was not determined in six samples; Sb was not determined in 31 samples; Mo was not determined in 33 samples; Ag was not determined in 36 samples; Cd was not determined in 107 samples; Sn was not determined in 157 samples; Te was not determined in 46 samples; Bi was not determined in 44 samples; whereas Hg was not detected in any of the studied wine samples out of 180.

All of the proposed methods are sensitive toward the analyzed elements and allow the adequate and almost simultaneous determination of the studied elements. The results of the mineral composition measurements are summarized in Table 5 and Figure S1 (Supplementary Materials) and presented in the form of basic statistics such as median, mean (expressed as mean value for the three measurements performed for each sample), standard deviation, min and max values. The results for Hg were not included since no Hg was detected in the studied samples. What can be observed is a relatively high dispersion of the results depending on the studied element, which, in general, stayed in agreement with the literature data [28,33,34]. The average contents of the median values for the elements in the wine samples decreased in the following order: K > Mg > Ca > B > Fe > Mn > Rb > Sr > Zn > Ba > Ti > Cr > Cu > Pb > Ni > Li > Co > Mo > Ag > Bi > Sb > Tl > U > Be > Te > Sn > Cd > Hg. This rank corresponds to the divisions made in Table 2 with regard to the levels of elemental components commonly found in commercially available wines. According to the concentration ranges summarized in Table 2, the group with the highest levels of elements such as K, Ca, and Mg can be found. In the next group, we could distinguish elements like B, Fe, Cu, Mn, or Zn. Slightly lower values were reported for Rb, Sr, or Ti. In the next group, we can distinguish Ba, Pb, Cr, Li, or Sn. About one order of magnitude lower values were reported for metals like Co, Mo, and Ag. The lowest values were typical for metals such as Sb, Be, Cd, Hg, Ta, Tl, or Bi. In another study by Rodrigues et al. (2011) [1], a similar tendency could also be observed for Portuguese wines. In this work, the ICP-MS technique was employed to determine 17 elements (Al, As, B, Ba, Ca, Co, Cu, Fe, K, Mg, Mn, Na, Ni, P, Pb, Sr, Zn) in a total of 85 wine samples (47 white wines and 38 red wines). According the obtained concentration range in wine samples (based on the median value as a central line), the elements showed the following order: K > P > Mg > Ca > Na > B > Fe > Mn > Al > Zn > Sr > Ba > Cu > Ni > Pb > As > Co. This order quite well represents the relations in the element content obtained in our study.

Due the fact that the authors in many papers have used the mean as a central value (not the median value), we decided also mostly to employ the mean as a central line indicator instead of the median for the comparisons proposed below (with some exceptions in few cases). 

#### 3.1.1. Cd and Pb

In our study, the cadmium content was higher than the maximum concentration permitted according to the OIV standard (Table 1) for eight samples (three red, five white, and one rose). The maximum concentration of Cd obtained in this work (31.04 µg/L) was recorded for dry white wine coming from France, Gascogne with an alcohol content of 10.5% and the grape varieties Colombard, Gros Manseng, Sauvignon Blanc, and Ugni Blanc (the 55W sample). However, if we compare the obtained results with other current standards in different countries that are less stringent (e.g., in Australia, the admissible level for Cd is as much as 0.05 mg/L), the maximum level was not exceeded for any of the samples.

For lead, the maximum concentrations against the OIV standard (Table 1) were exceeded in the case of nine samples (three red, two white, and four rose). The highest content of Pb (597.6 µg/L) was observed for dry red wine from California, USA, with an alcohol content of 15% and the grape varieties Zinfandel, Petite Sirah, Merlot, and Malbec (the 18R sample). Slightly lower standards for Pb (0.02 mg/L) were provided by the Commission Regulation (CE) and Polish internal regulation given by Ministry of Health. After comparing the results with the maximum permissible concentration given by above-mentioned institutions, the level of Pb was exceeded only in three cases.

The concentration ranges obtained in this research for cadmium and lead (which are quite regularly determined in wine from the safety of consumption perspective) were as follows: <LOD–31.04 µg/L (Cd) and <LOD–597.6 µg/L (Pb). Compared to the work of Płotka-Wasylka et al. (2017) [25] (<LOQ–18.4 µg/L (Cd) and <LOQ–118.3 µg/L (Pb)), we obtained slightly higher values. Taking into account the mean values in this paper, the levels of Cd (<LOD) and Pb (50.28 µg/L) were higher only in the case of lead when compared to the work of Kim et al. (2004) [26], where the mean value for Cd was 0.5 µg/L and for Pb was 29 µg/L. Similar results were gathered in the work of Serug et al. (2008) [27] (mean value of Cd: 0.13 µg/L; mean value of Pb: 9.5 µg/L), where quite low values when compared with our study were obtained. High results of cadmium or lead concentrations can be disturbing, however, in the literature, there are many reports on exceeding the permissible standards. According to the comparison of the content of certain elements in wines from different countries presented by Woldemariam et al. (2011) [19], the limits for cadmium were exceeded in the case of wine from Spain (max content 19 µg/L) and Hungary (max content 54 µg/L), whilst for lead, the maximum permitted content according to the OIV was exceeded for wines from the Czech Republic (max content 1253 µg/L), Italy (max content 350 µg/L), and Ethiopia (max content 310 µg/L). In this study, the permissible limit of Cd was exceeded for wines from the USA, Poland, Bulgaria, France, Italy, Greece, and Austria. In case of Pb the limit was exceeded for wines from the USA, Poland, Slovakia, Australia, and Italy in relation to the OIV standard. The fact that the results exceeded the norms for Cd and Pb in over 60% of cases concerning dry wines is interesting. Some authors have indicated that higher concentrations of these metals are probably the result of the infractions of technological norms, possible pollution from the container, or as a consequence of mixing different kinds of grape harvest [35]. The main reason for Cd contamination of agricultural soils is the application of phosphate fertilizers. In the case of enological source, contamination may occur at different stages of wine production. Moreover, in some reports, higher levels of Cd and Pb can be found in wines from vineyards located close to road traffic or situated in industrial areas [35]. As mentioned in the introduction, possible sources of heavy metals are the geochemical composition of soil, anthropogenic sources such as environmental pollution, and agricultural treatment by fertilizers, pesticides, and fungicides. Unfortunately, wine composition can also be affected by heavy metals like Cd and Pb delivered from wine production, wine processing, conservation, and bottling, especially that during the wine making process, the grape material is often in long contact with various materials such as stainless steel, wood from a variety of barrels, glass bottles, and others [36].

#### 3.1.2. Cu

The level of copper for only one sample was exceeded (1071 µg/L) according to the OIV standard (Table 1). This sample was dry red wine from the Barossa Valley, Australia, with an alcohol content of 14% and the grape variety Syrah (27R sample). If we consider the other standards for Cu available in Table 1, all analyzed wine samples were within the acceptable limits. For copper, the results were in the following range of 4.93 to 1071 µg/L. Generally, this range stayed in agreement with the data obtained in other studies [2,5,19,28]. Apart from contact with copper or bronze materials during the winemaking process, the major sources of wine contamination with this element are pesticides [34,36]. Cu in wine can originate from the so-called Bordeaux mixture of copper sulfate (CuSO_4_) and calcium hydroxide (Ca(OH)_2_) solution used as a fungicide in vineyards to protect against downy mildew, powdery mildew, and other fungi. This mixture can be used sometimes in elevated amounts, resulting in the accumulation of this element in soil. For this reason, this mixture was officially banned from the European community [36].

Furthermore, copper is frequently added to wine as it eliminates smelly characters associated with organic sulfur compounds that can form during fermentation and bottle aging [35,37]. On the other hand, it is thought that elements like Zn, Cu, and Fe can have a direct influence on the wine quality. Elevated copper concentrations higher than 1 mg/L can contribute to a metallic bitter taste and might generate turbidity. For this reason, during the production of wine, the levels of this element are normally kept below 0.5 mg/L [36].

#### 3.1.3. Ti

In the case of Ti, the concentration of this metal in the studied wine samples was not exceeded against the German standards (in OIV regulations, this metal is not included). For titanium, we obtained slightly higher mean values compared to the work of Płotka-Wasylka et al. (2018) [5] (mean value for white wine was 33 µg/L; mean value for red wine was 30 µg/L [11]). However, Bentlin et al. (2011) [21] indicated in his work that the mean values for Ti were very close to those obtained in this paper. Depending on the country of origin of the wine, it was 137 µg/L (Argentina), 126 µg/L (Brazil), and 143 µg/L (Chile and Uruguay), whilst we obtained mean values of titanium at the level of 108 µg/L. Ti can be treated as a fingerprint of soil contribution. This metal can also be employed as a additive for bottle coloring.

#### 3.1.4. Zn

The last metal indicated in the OIV standards and for which the admissible levels are given is Zn. For any of the studied samples, the admissible level was exceeded. For this element, the range of obtained values was: <LOD–4046 µg/L, while in other studies, the maximum concentration was 316 µg/L [25] and 670 µg/L [2], respectively. On the other hand, the mean value in this paper was 250.4 µg/L, which is a similar value to the one reported by other authors, and reached 640 µg/L [27].

It should be noted that Zn is a trace element naturally found in soil. For this reason, Zn plays a key role in the growth of plants [38].

However, there are no regulations regarding the permissible concentrations for many other elements in the wines analyzed in this study such as Cr, Mn, Ba, Bi, Ni, etc.

#### 3.1.5. Li

The lithium concentration in this work varied between <LOD–1325 µg/L. However, when the median values were compared for this element with the literature data, our results seemed to be similar to the level of Li obtained by other authors [5,22,23].

#### 3.1.6. Cr 

Maximum concentration of Cr in this work was 536.8 µg/L.

Maximum concentration of Cr obtained in this work was 536.8 µg/L. In other study the concentrations for Cr reported by authors were within the range of 3–1728.8 µg/L [6,30,31]. 

#### 3.1.7. Rb

The results obtained for rubidium also corresponded to the literature data. Irina et al. (2013) [6] determined a median value of Rb in 60 samples of wine from Romania at the level of 781.45 µg/L, where in our case, it was 658.6 µg/L.

#### 3.1.8. Mn

The mean manganese value obtained in this paper was 910.1 µg/L. This result was also within the ranges indicated by other researchers [2,5,6,23].

#### 3.1.9. Fe

The range of the results obtained in this work for iron was 154.9–10250 µg/L, which was comparable with the outcomes presented by Banović et al. (2009) [28] (810–6200 µg/L). On the other hand, in the work of Płotka-Wasylka et al. (2017) [25], the range of obtained results for Fe was <LOQ–969 µg/L. Nevertheless, there was no consensus in all cases.

The presence of Fe is correlated with the characteristics of the soil as well as the equipment and the stabilization treatments [38].

#### 3.1.10. Ni

Exceptionally good agreement was obtained for the nickel results. The average value in this study was 40.59 µg/L, whereas other researchers have indicated the following mean values for Ni of 53 µg/L for red and 64 µg/L for white wines [5], 55.15 µg/L [1], and 60 µg/L [31]. Ni and Zn are elements essential for vegetal growth and it is likely that their contents in wine derive from plant uptake from soils. They can both be present in soil as a consequence of metal contamination and due to the use of certain pesticides and phytosanitary products that may be applied to protect the plants from harmful organisms or to prevent their effects and eliminate unwanted plants. It was also proven that Zn and Ni are among the elements that may have an increased concentration due to effective contamination (e.g., by contact with stainless steel containers during the vinification) [1].

#### 3.1.11. Co

A slightly higher mean value than that for Ni was obtained for cobalt. In our work, it was 14.41 µg/L, while Płotka-Wasylka et al. (2018) [5] obtained a mean value of 2 µg/L.

#### 3.1.12. Mo

In our case, the mean value of Mo was much higher (2379 µg/L) than the value reported in another study [5], but the median value significantly differed from the mean (6.21 µg/L), which better reflected the cross-section of all results. According to the mentioned report, the determined mean level of molybdenum was 7.4 µg/L for white and 3.4 µg/L for red wines [5].

#### 3.1.13. Ag

The median value of the silver content determined in a given set of samples was 3.34 µg/L. This is in line with the work by Irina et al. (2013) [6], where the median value for 60 samples of white and red wines was 7.44 µg/L.

#### 3.1.14. K

In the case of potassium, the values obtained in this study were in the range between 0.48 and 1047 mg/L with the mean value equal to 156.7 mg/L, which is in line with the values obtained by other researchers [19,25].

K is treated as the main positive ion in wine by many authors. The content of K can be greatly varied with the variety of grapes, soil, and climatic conditions, time of harvest, the temperature of fermentation and storage, and the pH and the use of ion-exchange resins [38].

#### 3.1.15. Mg

In this study, a relatively wide range (13.17–611.6 mg/L) in the obtained results was noted for Mg with a mean value reaching 85.76 mg/L.

In the work of Woldemariam et al. (2011) [19], magnesium was determined in four different brands of wine produced in Ethiopia. The content of this element was in the range between 58.1 and 79.2 mg/L. On the other hand, Płotka-Wasylka et al. (2017) [25] determined the magnesium concentration in the following range from 5 to 19.2 mg/L, however, in this case, the conducted analysis concerned only homemade fruit wines. Gathered in our work results suggest better compatibility with the work of Woldemariam et al. However, in our case, the number of wine samples analyzed was much greater, which may highly influence the variability of the data set.

Authors suggest that magnesium content in wines is correlated with the natural Mg content of grape berries. Moreover, Mg along with Mn are recognized as mobile elements in soils, thus they are major micronutrients for plants and grape berries [38]. Some studies have revealed that Mg content is dependent on its concentration in vineyard soil, and that the contents of Mg, Mn, Ba, and Sr are mutually dependent. These metals have been categorized by many authors to the group of lithophile elements and it is likely that their origin in wines is entirely or mostly derived from the vineyard soils [1].

#### 3.1.16. Ca

Comparing our results for calcium with the already mentioned studies [19,25], it can be seen that other researchers have obtained slightly lower values. However, taking into account the mean value from this study (84.78 mg/L), we also have the same order of magnitude. On the other hand, Fabian et al. [3] indicated the median for calcium in wines from three production regions in Argentina at the following levels: 50 mg/L (Córdoba), 31 mg/L (La Rioja), and 283 mg/L (San Juan), while the median calcium content in this study was 66.24 mg/L. Taking into account the huge variety of samples in this study compared to the mentioned author, we obtained, without a doubt, a very good agreement.

Calcium is thought to be a natural component of berries [38]. Moreover, Ca along with Al and Fe are elements that seem to be quite abundant in soils. Due to their high levels, the potential differences between wine regions can be successfully masked [1].

#### 3.1.17. U

Exceptionally good agreement was obtained for the uranium results compared with the last mentioned author. Again, depending on the country of origin of the wine, the mean values were 1.2 µg/L for Argentina, 0.1 µg/L for Brazil, 0.4 µg/L for Chile, and 0.7 µg/L for Uruguay. The range of our results was <LOD–2.345 µg/L with a mean value of 0.408 µg/L.

#### 3.1.18. Sb

Antimony is the next element for which we obtained very good agreement with the results published by Płotka-Wasylka et al. (2018) [5] (mean value of 2.3 µg/L for white wines and 0.4 µg/L for red wines) and Bentlin et al. (2011) [21] (mean value of 0.249 µg/L), while in our study, the mean value reached 1.469 µg/L.

#### 3.1.19. Sn

Regarding the Sb content and also for Sn, very good agreement with the work of Bentlin et al. (2011) [21] was achieved. The average value for Sn in our study was 15.20 µg/L, while the aforementioned authors determined the mean tin levels in 53 wines from South America at the level of 17.83 µg/L.

#### 3.1.20. Sr

The content of strontium found in our study had a relatively wide range between 95.99 and 3033 µg/L. This again stayed partially in line with the work by Irina et al. (2013) [6] where the range of obtained results was 144.34–1600 µg/L. However, in our work, a larger number of samples, which were quite diversified in terms of their origin and type, were analyzed when compared to the mentioned paper. On the other hand, considering the mean value (in our case it was 605.2 µg/L), really good agreement was obtained with the work of Bentlin et al. (2011) [21], in which mean value reached 570 µg/L.

#### 3.1.21. Bi

Considering the results obtained in this work for bismuth (mean value 12.4 µg/L), they were in some agreement with the work of Płotka-Wasylka et al. (2018) [6] (mean value for white wine 49 µg/L; mean value for red wine 2 µg/L [11]). 

#### 3.1.22. Ba

Zioła-Frankowska et al. (2017) [23] obtained the mean value for Bi where three different sample preparation methods were included in the range of 119–285 µg/L. This is in line with the mean value evaluated in this paper of 125.9 µg/L.

#### 3.1.23. B

For an element such as boron, no agreement was found with the literature data. In the works of Płotka-Wasylka et al. (2018) [5] and Zioła-Frankowska et al. (2017) [23], the mean results were 3730 mg/L for white and 6870 mg/L for red wines [6] and in the following range of 2.441 to 5.258 mg/L [23]. In turn, Coetzee et al. (2005) [22], after examining 40 wines from three regions of South Africa, obtained insignificantly lower values for boron than in the mentioned studies ranging from 2.55 to 3.50 mg/L, while in this study, the mean value for B was 16.08 mg/L.

B along with K are essential nutrients for plants and their related contribution is likely to be associated with fertilization practices that may vary between different vineyards and plant uptake processes [1].

#### 3.1.24. Te

The tellurium concentration determined in this study was in the following range of <LOD–3.913 µg/L. The literature data indicated a similar concentration of this element. Pérez-Trujillo et al. [24] determined the Te content in 153 wine samples at the level of 0.1–0.43 µg/L.

#### 3.1.25. Be

As Korkisch et al. [29] pointed out, Be is recognized as one of the trace elements that is either not contained in wines or cannot be detected. However, the authors in that study stated the presence of beryllium in wine samples in the range <LOD–0.15 µg/L. They also found that wines originating from the same locality contained very similar Be concentrations. In the set of samples analyzed in this paper, the concentration of Be was in the range of <LOD–0.343 µg/L, where the median value was 0.021 µg/L.

#### 3.1.26. Tl

In this work thallium was marked within the following range <LOD–1.221 µg/L (mean value 0.721 µg/L and median 0.360 µg/L). This is a slightly wider range than that obtained at work Cortezee et al. (2005) [22]. On the other hand, the mean values for Tl in white (0.87 µg/L) and red (0.81 µg/L) wines given by Płotka-Wasylka et al. (2018) [5] are comparable with the results in our paper. 

#### 3.1.27. Hg

Analysis of the mercury content was performed using an automatic mercury analyzer by the cold vapor AAS technique. The data showed that this element was not present in all of the 180 wine samples (all results were below the limit of quantification).

Płotka-Wasylka et al. (2017) [25] drew similar conclusions in terms of Hg level in wine samples. The authors analyzed 17 homemade fruit wines. The content of toxic Hg in most of the samples was below the limit of detection. Only in one of the samples it was possible to determine the total mercury content with the CV-AAS method and this level reached 0.437 µg/L. The same authors also analyzed 44 wine samples in another work [5] using the ICP-MS technique. However, in that case, they obtained results of Hg concentration for all tested samples above the limit of detection (0.31–0.51 µg/L), which is quite surprising. However, it needs to be noted that all metal content including Hg in the wine samples was below the maximum concentrations given by the OIV.

### 3.2. pH Measurement

All the wine samples were tested against pH value, which was treated as an additional variable for potential sample discrimination and categorization. Averaged results for pH measurement of all tested samples were analyzed by STATISTICA 10 software. Table 6 presents the basic statistical parameters of the results obtained for all wine samples depending on the type of wine.

The normality of the distribution for all pH mean values was tested. Gained *p*-value for an established significance level of 0.05 implied that for the mean pH of all wine samples, the outcomes followed the normal distribution. On the other hand, the analysis of pH in individual groups of wine types (red. white and rose) showed that the normal distribution was achieved in the case of white and rose wines, whilst for red wines, the opposite conclusion can be drawn (Table 6). The existence of statistically significant differences in the mean pH value between red and white wines and between red and rose wines was also revealed.

**Figure 2:**
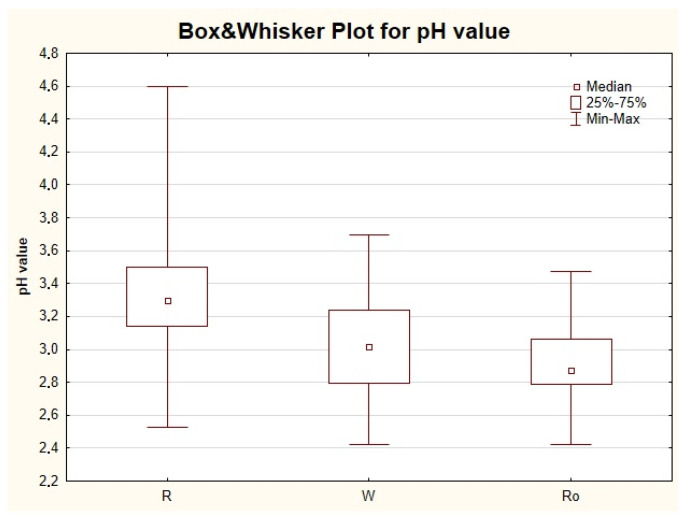
Box and whisker plot of pH value obtained for red (R), white (W), and rose (Ro) wines.

The mean pH value for red wines was 3.33, white wines reached 3.01, and for rose, it was 2.89 (Figure 2). The higher pH level for red wines is due to the fact that a second fermentation process is carried out for this type of wine (malolactic fermentation, MLF). This is typical only for red wines, while white (with some exceptions where it is expected to neutralize any perceived acidity) and rose wines do not undergo this process. It gives red wines a less acidic character compared to white or rose wines and an increase in microbial stability due to the removal of malic acid [39,40]. Malolactic fermentation is generally conducted immediately after alcoholic fermentation (less frequently in the course of) with the addition of Oenococcus oeni for the transformation of more aggressive diprotic malic acid to milder lactic acid (single protic group). This process also has an impact on the sensory characteristics of wines. What should be noted is the fact that organic acids present in wine and retarded from the grapes do not disappear in the vinification process. Sugar reacts with yeast to give alcohol, but the acidity of products remains. Furthermore, during the vinification process, new acids may occur like volatile acetic acid. It is formed when yeast cells produce its trace amounts as a result of the activity of Acetobacter bacteria, which in an aerobic environment converts ethanol into acetic acid. The acids that are originally present in wine are malic (and the derivatives—malates), tartaric (and a derivative—potassium bitartrate), and, in trace amounts, citric acid. Their levels will depend upon the grape variety, the degree of maturity of the grapes (especially for malic acid since fruit harvested too early will have a higher malic acid content than fruit harvested at the right time), and the climate. The greater maturity of the grapes reduces the malic acid and increases the sugar content. Therefore, the warmer the climate or the later the harvest is planned, the more sugar will develop in the bunches and somehow balance the acidity of the product. Thus, in regions with a relatively cold climate, dry wine can be achieved by the conversion of an excessive amount of malic acid into lactic acid by malolactic fermentation initiated by lactic acid bacteria [41].

In our study, the univariate analysis of variance was additionally applied to test possible differences among various categories of wines (dry, semi-dry, sweet, and semi-sweet). The results revealed that dry wine (median 3.19) and semi-dry samples (median 3.21) can be characterized by higher pH values than sweet wine (median 2.98) and semi-sweet wines (median 3.03). Statistically significant differences were stated between pH values for dry wines (group with the highest number of samples and biggest variation of the results) and semi-sweet wines (the group with the lowest number of representatives). Additionally, among only red wines (represented by the highest number of samples when compared to the other types of wine) for which only one grape variety was identified, again the univariate analysis of variance was performed. Among the nine identified grape varieties, no statistically significant differences were stated against pH values. The highest median value (3.39) was reported for Merlot-based wines, while the lowest was for Riesling (2.86). It is assumed that dry Riesling has a higher content of tartaric and malic acid than dry Chardonnay (in our study median value reached 3.01) grown in the same climate.

Rodrigues et al. (2011) [1] observed a similar dependence as was indicated in our study. In this work, a total of 85 wine samples (47 white wines and 38 red wines) were analyzed originating from four Portuguese regions. The pH values obtained in this work varied between 3.0 and 3.9. However, the employed univariate analysis of variance revealed that the pH for white wines (mean of 3.4) was significantly lower when compared to red ones (mean of 3.6). The authors also used the analysis of variance to potentially discriminate samples among the studied regions from which the wine samples were collected, but no statistical significant differences in pH between the regions for red wine nor for white wine were stated.

### 3.3. Elemental Analysis for Wine Type

As already mentioned, the information about the content of some elements, mainly heavy metals (Pb, Cd), is essential in terms of the unfavorable impact on the human body. On the other hand, some elements are used as indicators, which can be especially helpful in wine categorization.

Preliminary analysis of the concentrations of the determined metals allowed us to find some dependence between the content of individual elements and the type of wine. Basic information (mean and median value) on the analyzed metal concentration (µg/L) for red, rose, and white wines is given in Table 7. Higher content (simultaneously for the mean and median value) was recorded for white wines (when compared to red and rose wines) for elements such as Ag, Be, Bi, Cd, Co, Li, K and Ti. Red wines were characterized by higher values of mean and median of B, Ba, Cr, Cu, Mn, Sr and Zn in contrast to other wine types (white and rose). In turn, the highest Fe and U content was recorded for rose wine samples. Płotka-Wasylka et al. (2018) [5] also grouped the studied elements in wines based on their levels in relation to the type of wine. According to this study for elements such as Ag, Al, As, Bi, Cu, Sb, Se, Sn, Zr and Zn much higher levels were noticed for white wine samples than for red ones. In the case of elements like B, Ba, Fe, K and Mn their higher content was found in red wine samples. Thus, it can be stated that based on the results of both researches higher levels of Ag, Bi and Zn in white wines and higher amount of B, Ba and Mn in red wines were confirmed. These similarities seem to support the general conclusions drawn by Greenough et al. [19]. According to these authors the chemical composition within particular wine type is thought to be consistent within the group (red or white). As a consequence it was pointed out that the location of grape cultivation plays a key role, not the age of wine or the grape variety. These findings stays in agreement with other works in which a suggestion that the presence of some elements is influenced by the type of wine is made [5]. Also other authors implied that wines of a certain type are similar in terms of their chemical composition [7]. 

In the next stage of data evaluation the potential statistical differences in the level of studied metals were tested. For this purpose Kruskal–Wallis test was employed. It was revealed that when considering the studied set of samples in terms of wine type, the existence of statistically significant differences in the concentration of the following elements can be found: Ba, Ca, Cr, Cu, Fe, K, Mn, Sb, Sr, Ti and U. In the case of all mentioned elements where the existence of statistically significant differences was demonstrated, the level of significance (*p*) was less than 0.05.

For Cr and Cu, the existence of statistically significant differences between white and red wine was proven. For both elements much higher levels of these metals (median and mean values) were stated in red wines when compared with white ones. In the case of Fe, statistically significant differences in the level of this metal in red and rose wines were stated. The observed statistically significant differences in the content of Mn, Ba, U, Sb, Sr, Ti, and Ca were related to the following pairs: white–red and rose–red wines. For K, the existence of statistically significant differences were confirmed for pairs of red–white and white–rose wines. It can be summarized that in our study rose wines can be differentiated from the rest of studied samples based only on the content of two elements (Fe and U). They much higher levels (in terms of mean and median values) seem to be quite characteristic for rose beverages. For these two metals statistically significant differences were observed between the flowing pairs: red and rose wines (for concentrations of Fe and U) and between white and rose wines (only for concentration of U). For white wines the elevated concentration of Ti when compared to red wines can be noted. Moreover, white wines can be differentiated from both red and rose wines based on the K levels, which were significantly higher in the white wines group. For red wines much higher concentrations were determined for elements such as Cr and Cu (when compared with white wines group) and Mn, Ba, Sr (in contrast to the both white and rose wines groups). These elements can be potentially used to differentiate red wines from the rest of samples (based on the Mn, Ba and Sr levels) or form the white wines (in reference to the Cr and Cu concentrations). Even though some statistically significant differences can be stated in the Ca and Sb levels between white and red wines and red and rose wines no conclusive conclusions can be drawn from the concentrations of these metals. For these two elements the highest values were reported in the white wines group, while the highest median values were observed for the rose wines group. All basic statistical information regarding the concentration of Cr, Mn, Cu, Ba, and Fe is given in the Supplementary Materials (Figures S2–S12, Tables S2–S12). 

### 3.4. Elemental Analysis for Country of Origin

It has been proved that the composition of soil is directly related to the content of individual elements in wines and can be somehow linked with the country of production [42]. Therefore, the concentration of certain metals is also used by scientists to identify the origin of alcohol beverages in terms of their authenticity. Many studies have been performed in this field, however, there is still a need to deliver some current data, which can not only expand our knowledge, but can also be used in simulation models. 

Based on the many studies, the elemental composition of wine is mostly related to its geographical origin [3,7,21]. Many authors have also suggested that suitable discriminant analysis can accurately classify the wines according to the vineyard [7]. Thus, a very important aspect of this work was to establish the relationship between the content of selected elements and the origin of wine. On the basis of the Kruskal–Wallis test, the existence of statistically significant differences in the concentration of the following elements was demonstrated: Be, Co, Mn, Pb, Rb, Sb, Sr, U and Zn. In the case of all mentioned elements the existence of statistically significant differences was confirmed based on the the level of significance (*p*), which was less than 0.05. For beryllium, the differences concerned wines from Poland (median value <LOD) and Spain (median value 0.085 µg/L). For cobalt, only in the case of Poland (median value 3.621 µg/L) and France (median value 22.53 µg/L) were statistically significant differences noted. For manganese, these were wines from Poland (median value 314.9 µg/L) and the USA (median value 1069 µg/L), Poland (median value 314.9 µg/L) and France (median value 862.6 µg/L), Poland (median value 314.9 µg/L) and Italy (median value 805.9 µg/L) as well as Poland (median value 314.9 µg/L) and Australia (median value 2179 µg/L). The differences in rubidium concentration were significant for a pair from Poland (median value 329.5 µg/L) and Portugal (median value 1653 µg/L), while for lead, it was wine from Poland (median value 329.5 µg/L) and Slovakia (median value 1865 µg/L). For strontium, the differences concerned wines from Poland (median value 288.5 µg/L) and the USA (median value 874.3 µg/L), Poland (median value 288.5 µg/L) and Italy (median value 613.0 µg/L), Poland (median value 288.5 µg/L) and Spain (median value 890.6 µg/L), Poland (median value 288.5 µg/L) and Australia (median value 2123 µg/L) as well as Australia (median value 2123 µg/L) and France (median value 343.6 µg/L). For antimony, these were wines from Poland (median value 0.815 µg/L) and France (<LOD), France (<LOD) and the USA (median value 2.147 µg/L), the USA (median value 2.147 µg/L) and Italy (median value 0.275 µg/L), the USA (median value 2.147 µg/L) and Portugal (median value 0.113 µg/L), the USA (median value 2.147 µg/L) and Spain (median value 0.202 µg/L), and the USA (median value 2.147 µg/L) and Australia (median value 0.065 µg/L). In the case of uranium, the existence of statistically significant differences were observed for France (median value 0.181 µg/L) and the USA (median value 1.175 µg/L), the USA (median value 1.175 µg/L) and Italy (median value 0.178 µg/L), the USA (median value 1.175 µg/L) and Australia (median value 0.057 µg/L) as well as Poland (median value 0.298 µg/L) and Australia (median value 0.057 µg/L). In turn, for zinc, statistically significant differences concerned wines from Poland (median value 30.53 µg/L) and the USA (median value 296.2 µg/L) and from Poland (median value 30.53 µg/L) and Australia (median value 441.2 µg/L). The most important statistical information connected with the division of samples against the country of origin is included in the Supplementary Materials (Figures S13–S21, Tables S13–S21).

What should be highlighted is the fact that for all elements for which the existence of statistically significant differences was confirmed, wines originating from Poland were listed in most of the comparisons. Although wine samples from Poland were represented by the largest number of samples (42), in the case of e.g., Zn, Mn, and Be, the lowest median values were obtained among all the studied groups. With an exception of Sb and U, for the rest of the mentioned elements (Be, Co, Mn, Pb, Sr, Rb, Zn) Poland was characterized by the lowest median value in the indicated pairs of countries. This suggests that few elements can be potentially applied as the useful indicators for a country of origin for samples produced in Poland. Moreover, relatively low levels of many of studied elements in Polish wines should be treated as a beneficial information from the consumer point of view. Only for some metals considerably high variation of results within 25% of the highest values was noticed, like for Pb, Zn and Mn. In all these cases the wines from Poland included in this analysis originated from different regions of Poland, were produced by different producers but based on the same grape variety (Johanniter), which can partly explain the stated differences. 

In our opinion, based on the gathered results also products from USA and Australia can be successfully discriminated from the rest of wines. For USA the most characteristic metal for positive identification of the country of origin seems to be uranium, whereases for Australia – strontium and manganese. As proven, uranium is very easily transferred from soil, rocks, or uranium waste dumps and accumulates in vegetation or in water. Different plants accumulate uranium to a different degree. As a rule, sugar-, starch-, and fat-rich foodstuffs have proven to be uranium-poor (fruits, seeds, flour), whereas leafy vegetables, tea, and herbs can be uranium-rich. The uranium contents of traditional German beverages vary between 0.30 µg/L in beer and 1.3 µg/L in white wine. It was found that the uranium content in the water used for the production of beer, and other beverages seems to play a key role. The calculation of the uranium intake of omnivores showed that the major source of this element (approximately 40%) came from different beverages. In the case of men, 7% of their uranium intake is from alcoholic beverages [43]. As the USA is famous for its rich mineral resources, especially uranium ore, it is not surprising that wines made from vines growing in such soils have higher values of this element. The identification of uranium may be a discriminatory criterion for wines from the USA against wines from other countries as it was proven in our study. Uranium content (median and mean values) was the highest in products originating form the USA among countries for which more than one wine sample was collected. 

In turn, Australian wines were in this work characterized by much higher values of elements such as strontium and manganese (the highest median, mean and max values were obtained in this group). It should be mentioned as well that despite the fact that no statistically significant differences were stated for Cu concentrations vs. country of origin, for this metal the highest mean value and one of the highest median value (next to the wines from Chile) were noted for wines from Australia. In the case of all these elements, one of the key sources of their origin is the absorption from vineyard soil and used agrochemical treatments. The amount of metal ions transferred into grapes is influenced by the type of soil and the grape absorbance properties [44]. Characteristics for a hot, humid climate, in areas covered with equatorial and subequatorial forests and high grass savannas are laterite soils, covering most of Australia. These soils are characterized by a strongly acidic reaction at the surface level, the presence of a mainly brick-red color, and a strong concentration of elements such as aluminum, iron, manganese, and strontium [45]. 

Similar studies has also been performed all over the world in which the attempt was made to differentiate the samples according to their origin. In the research carried out by Coetzee et al. (2005) [22], the concentration of 40 different elements in 40 samples of wine from three important wine-producing regions: Stellenbosch, Robertson, and Swartland in South Africa was determined. The key to the correct classification of wines from each region was based on a stepwise discriminant analysis procedure, where linear combinations of the log-transformed element concentrations of Al, Mn, Rb, Ba, W, and Tl were selected. In this work Rb and Mn were selected as crucial elements in determining the real geographical origin of wine. In our study both these elements can be used for positive discrimination of Polish products from the rest. In turn, Fabain et al. (2010) [3] evaluated metal profiles in the context of geographical origin of Argentinean wines. Thirty-one samples of wine from three major wine-production regions were analyzed: San Juan, La Rioja, and Córdoba. It should be noted that samples were obtained directly from producers and none of the studied wines were fermented or aged in wood. The authors received good separation amongst wines from three different provinces. They stated that wines from these three regions of Argentina could be clearly differentiated considering their content of K, Fe, Ca, Cr, Mg, Zn, and Mn. Like in our study Mn and Zn appeared on the list of essential elements in the discrimination of wine samples with respect to origin. The authors also concluded that 86% of metal contents in wines could be explained by the composition of vineyard soil. The remaining 14% could be attributed to other factors such as climate, agricultural practices, pollution, grape maturity at harvesting, wine-making techniques, etc. In other work Bentlin et al. (2011) [21] analyzed 54 red wines obtained from common grapes of Vitis vinifera species from South America (Argentina, Brazil, Chile, and Uruguay). As discriminant elements, Tl, U, Li, Rb, and Mg were chosen. The authors stated that factors such as the type of yeast used, the way the vines are cultivated, knowledge of the winemaker that influences the way of making wine, storage form, and fertilizers used may have contributed to the differences among the wines. Among indicated by these authors metals, only for Rb levels statistically significant differences were stated in our study (for wines produced in Poland and in Spain). 

In the literature data, it can also be found that Cr and Fe have been indicated as potential markers of the origin of wines from European countries [46,47]. However, the content of iron in wine is related to soil composition as well as the technological processes involved in wine-production [47,48]. Furthermore, there are also studies that show that wines from grapes from the same vineyard tend to be most similar, regardless of the time of their storage or grape variety. This theory clearly states that thee ‘fingerprints’ of wine are relict soil signatures that survive metabolic and winery processing since the uptake of elements is primarily influenced by pH or complexing agents (quite characteristic parameters for soil chemistry).

### 3.5. Elemental Analysis for Grape Variety

Most of the studied beverage samples (*n* = 180) consisted of a mixture of various grapevines. For this reason, it was almost impossible to make an appropriate division. Therefore, only those samples for which the producer declared only one grape variety were further taken into account (*n* = 38), while the other samples were rejected from the analysis.

**Figure 3:**
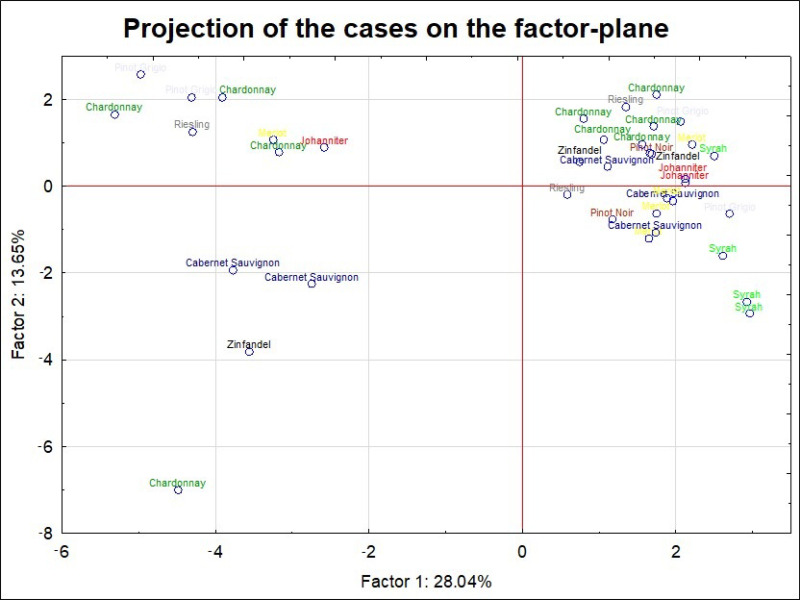
Projection of the cases on the factor-plane in 38 samples investigated in this study according to grape variety for the whole dataset [µg/L].

In this study, we distinguished nine grape varieties, respectively, Cabernet Sauvignon, Merlot, Pinot Noir, Syrah, Zinfandel, Pinot Grigio, Johanniter, Chardonnay, and Riesling (Figure 3). For a reduced dataset, the existence of statistically significant differences in the concentration of Cu, K, and Sr were proven (Figures S22–S24, Tables S22–S24 in the Supplementary Materials). For these metals the level of significance (*p*) for considered pairs was less than 0.05.

For copper, the differences concerned the Chardonnay and Syrah grape varieties. In the case of potassium, the existence of statistically significant differences were observed for Chardonnay and Cabernet Sauvignon. For strontium, the differences concerned pairs of grape varieties such as Syrah–Pinot Grigio, Syrah–Johanniter as well as Syrah and Riesling. It can be summarized that Syrah grape varieties can be characterized by the highest levels (mean, median and max values) of Cu and Sr, while Chardonnay grape variety by the relatively high amount of K. In the case of Syrah-based wine samples they all originated from the same country (Australia). As it was already indicated, the presence of high Sr levels can be an indicator of the origin for Australian wines. On the other hand, Syrah-based wines can be also characterized by much lower content of elements such as Ag, Cd, and Bi compared to others. Moreover, is the only grape variety that can be grouped into one cluster as a result of PCA analysis (Figure 3). Therefore, the observed grouping should be more related with the country of origin than the grape variety. 

In addition, five of the nine wine samples produced from the Chardonnay grape variety were grouped into a common cluster. These, in turn, are characterized by higher potassium content. It should be noted that in contrast to the Syrah variety, the Chardonnay-based grape varieties included in this analysis were produced in various countries e.g., France, Ukraine, Argentine, Australia, Bulgaria, Portugal, Moldavia. Thus, based on the available (highly reduced) set of samples, it is almost impossible to differentiate the studied beverages according to the grape variety other than Syrah, and partially Chardonnay. In general, a grouping of no more than two or three objects belonging to one grape variety was obtained (e.g., like for Cabernet Sauvignon or Johanniter).

Similar conclusion as in our study were drown by other authors. For instance, Greenough et al. (2008) [7], based on the results gathered for 17 white and 10 red wines from vineries from Canada, concluded that wines from grapes from the same vineyard tended to present similar chemical composition, regardless of the vintage, grape variety, or winery. This indicates that wine elements that can be called ‘fingerprints’ are relict soil signatures that survive metabolic and winery processing.

### 3.6. Chemometric Analysis of Multiparametric Data

The primary aim of the chemometric evaluation was to show the relationship between the wine samples in order to classify them according to the content of individual metals. The chemometric methods used were hierarchical cluster analysis and principal components analysis (PCA). A cluster dendrogram was performed after raw data standardization (squared Euclidean distances; Ward’s method of linkage).

**Figure 4:**
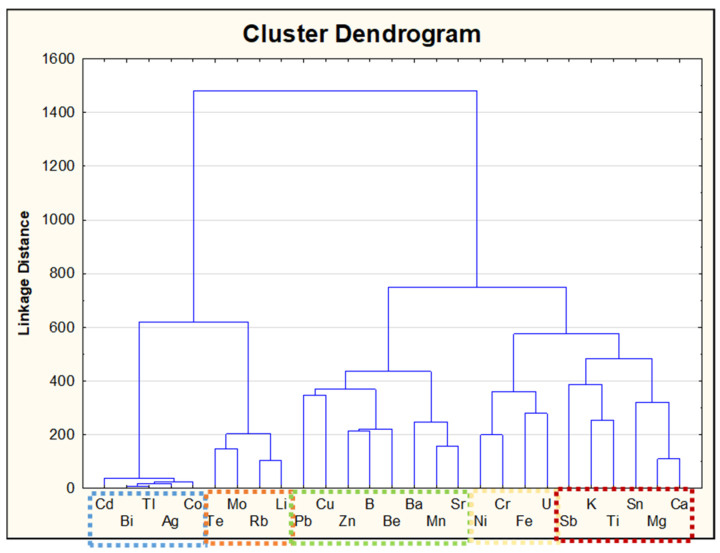
Hierarchical cluster dendrogram for 18 variables.

The HCA analysis (Figure 4) made it possible to select the following five clusters: C1 (Cd, Bi, Tl, Ag, Co), C2 (Li, Rb, Mo, Te), C3 (Pb, Cu, Zn, B, Be, Ba, Mn, Sr), C4 (Ni, Cr, Fe, U), and C5 (Sb, K, Ti, Sn, Mg, Ca).

In the next part of the data evaluation the PCA analysis was performed. Information on the factor loadings is presented in Table 8 and the projection of the variables is included in the Supplementary Materials in Figure S25.

Only after taking into account the first eleven components, we obtained over 80% of the explained variance. Performed PCA analysis revealed that the first two components explained about 36% of the total variation (Table 8, Figure S25 in Supplementary Materials). The first principal component (PC1) explained only 23.9 of the total variance. The PCA plot showed a high negative correlation between Co, Ag, Cd, Tl, and Bi, which corresponded integrally with the C1 cluster and Li, Mo, Te, which mostly correlated to the C2 cluster from the HCA method. PC2 explained almost 12% of the total variance and corresponded to the last element from C2 (Rb) and to four elements from C3 (Zn, Be, Ba, and B). In turn, PC3 indicated high factor loadings for two elements from C3 (Mn and Sr) and for two elements from C5 (Ca and Sn). The last element from cluster C3, Cu, was closely related to PC10. With the eight principal components, Pb was strongly correlated. The C4 cluster was dispersed among the remaining factors, respectively, where Cr and Fe presented the highest correlation with PC5, Ni with PC6, while U showed the highest correlation with PC7. The rest of the elements from the C5 cluster were related to the following principal components: Mg with PC4, Ti and K with PC5, and Sb with PC9. Part of the C5 cluster (Ti, Sn, Sb) corresponded to the configuration given by Płotka-Wasylka et al. (2018) [5] as the K4 cluster, which in turn can be called “soil toxic impact”. The same category was given to elements such as Cd and Pb in Cluster C4, some elements from Cluster C3 (B, Sr) and Mg, Ca as well as K from Cluster C5 corresponded partially with Cluster K3, K1 and K2, which the mentioned authors assigned to “soil specificity impact”. The PC11 factor explained only 2.9% of the variance and was related to Rb and Ba (however, these were not the highest values). Additionally, elements such as Ti, K, Ca, Mg, and Pb showed weak correlation with the two first components. Therefore, an attempt to eliminate these elements in order to increase the percentage of explained variance on the first two components was made. However, to obtain over 80% of the explained variance, it was necessary to reduce most of the analyzed variables. The projection of the variables on the factor plane for a reduced set of variables used the following elements: Cd, Te, Tl, Bi, Co, Rb, Mo, Li, and Be. In this case, variables such as Te, Mo, Rb, and Li were strongly correlated and similarly negatively related to the first and second components. Tl, Bi, and Co showed a very strong correlation with each other. Additionally, these elements were strongly negative related to the first and positive to the second principal component (Figure S26, Supplementary Materials).

## 4. Conclusions

The average contents of the median values for the elements determined in this study decreased in a similar order as suggested by the literature data. Thus, it can be stated that the ranges of particular elements seem to be characteristic for wine products. Moreover, the results obtained for all studied metals were, in general, in a good agreement with the values reported by other authors.

Comparing the obtained quantitative results for Cd, Pb, and Cu with the maximum concentrations established by the OIV standard, it can be noted that the admissible levels were exceeded in eight samples for Cd, in nine samples for Pb, and in one sample for Cu. In the case of Ti and Zn for any of the studied samples the obtained results were higher than the established permissible levels. What should be noted is the fact that there are no regulations regarding the permissible concentrations for many other elements in the wines analyzed in this study such as for Cr, Mn, Ba, Bi, Ni, etc.

Based on the analysis of mean and median values for studied 28 elements, it can be concluded that depending on the type of the wine, the level of the individual metals was different. Higher content (simultaneously for the mean and median value) was recorded for white wines (when compared to red and rose wines) for elements such as Ag, Be, Bi, Cd, Co, Li, K and Ti. Red wines were characterized by higher values of mean and median of B, Ba, Cr, Cu, Mn, Sr and Zn in contrast to other wine types (white and rose). In turn, the highest Fe and U content was recorded for rose wine samples. Moreover, it was revealed that when considering the studied set of samples in terms of wine type, the existence of statistically significant differences in the concentration of the following elements can be found: Ba, Ca, Cr, Cu, Fe, K, Mn, Sb, Sr, Ti and U. 

The results also revealed that dry wine and semi-dry samples can be characterized by higher pH values than sweet wine and semi-sweet wines. The higher pH level for red wines is due to the fact that a second fermentation process is carried out for this type of wine (malolactic fermentation). The highest median value of pH (3.39) was reported for Merlot-based wines, while the lowest was for Riesling (2.86). It is assumed that dry Riesling has a higher content of tartaric and malic acid than dry Chardonnay grown in the same climate. 

Although wine samples from Poland were represented by the largest number of samples (42 out of 180), in the case of e.g., Zn, Mn, and Be, the lowest median values were obtained among all the studied groups. Relatively low levels of many of studied elements in Polish wines should be treated as a beneficial information from the consumer point of view. Moreover, this study revealed that also wine samples from USA and Australia can be potentially discriminated from the rest of studied wines. For USA the most characteristic metal for positive identification of the country of origin seems to be uranium, whereases for Australia–strontium and manganese.

Based on the highly reduced set of samples, it was not possible to differentiate the studied wine products according to the grape variety other than Syrah, and partially Chardonnay. Since all the Syrah-based samples originated from the same country (Australia) thus, the observed grouping should be more related with the country of origin than the grape variety. 

## Figures and Tables

**Table 1:** The maximum admissible concentration of selected elements established by some countries in reference to standards given by the OIV [5,17,18,19,20].

Origin		Maximum Concentration of Metals [mg/L]			
	Cd	Cu	Pb	Ti	Zn
Australia	0.05	5.00	0.20	-	5.00
Germany	0.01	5.00	0.30	1.0	5.00
Italy	-	10.0	0.30	-	5.00
Poland	0.01	-	0.2	-	-
EC	-	-	0.2	-	-
OIV	0.01	1.00	0.15	-	5.00

**Table 2:** Inorganic constituents [mg/L] of wine (typical values of normal contents) [8].

1000–10	10–1	1–0.1	0.1–0.01	0.01–0.001	≤0.001
K 370–1120	B 5–2	Al 0.9–0.5	As 0.02–0.003	Co 0.02–0.001	Sb 0.006
Mg 60–140	Fe 1	F 0.5–0.05	Ba 0.3–0.04	Mo 0.01–0.001	Be 0.00008
Ca 70–140	Cu 0.5	I 0.6–0.1	Pb 0.1–0.03	Ag 0.02–0.005	Cd 0.001
Na 7–15	Mn 5–1.5	Rb 4.2–0.2	Br 0.7–0.01		Cs 0.0027
C 100–120	Si 6–1.5	Sr 3.5–0.2	Cr 0.06–0.03		Au 0.00006
P 130–230	Zn 3.5–0.5	Ti 0.3–0.04	Li 0.2–0.01		Hf 0.0007
S 5–10			Ni 0.05–0.03		Nb 0.001
Cl 20–80			V 0.26–0.06		Hg 0.00005
			Sn 0.7–0.01		Se 0.0006
					Ta 0.0005
					Tl 0.0001
					Bi 0.00015
					W 0.003
					Rare earths
					Radioactive elements

**Table 3:** Characteristics of tested samples in relation to their origin.

Country of Origin	Number of Samples			Grape Variety
	Red	White	Rose	
Italy	15	14	2	Sangiovese, Sagrantino, Merlot, Primitivo, Susumaniello, Nerello Mascalese, Nerello Cappuccio, Dolcetto, Pinot Grigio, Montepulciano, Barbera, Terrano, Negroamaro, Malvasia, Muller Thurgau, Pecorino, Garganega, Insolia, Cataratto, Grillo, Muscato
Poland	14	19	9	Johanniter, Riesling
France	9	5	2	Cabernet Sauvignon, Pinot Noir, Cabernet Franc, Cabernet Syrah, Merlot, Le Carillon, Grenache, Syrah, Chardonnay, Sauvignon Blanc, Ugni Blanc, Colombard, Rolle
USA	9	2	7	Concord, Ruby Cabernet, Zinfandel, Petite Sirah, Merlot, Malbec
Spain	8	6	1	Tempranillo, Pedro Ximenez, Listán Negro, Shiraz, Garnacha, Bobal, Garnacha Blanco, Sauvignon Blanc, Chardonnay, Verdejo, Merseguera, Moscatel, Xarello, Parellada, Macabeu, Gewurztraminer, Pinot Noir, Cava Rimarts
Slovakia	4	4	0	Alibernet, Cabernet Sauvignon, Trebbiano, Morawski Muscat, Muller Thurgau, Irsai Oliver
Australia	6	1	0	Cabernet Sauvignon, Merlot, Mataro, Syrah, Chardonnay,
Portugal	5	3	0	Castelao, Tinta Roriz, Alicante Bouschet, Touriga Nacional, Chardonnay, Loureiro, Arinto
Germany	0	4	1	Riesling
Bulgaria	2	3	0	Cabernet Sauvignon, Chardonnay
Hungary	1	3	0	Pinot Noir, Muscat Ottonel, Furmint, Chardonnay, Hárslevelű, Sárgamuskotály
Moldova	2	2	0	Cabernet Sauvignon, Merlot, Chardonnay
Chile	3	1	0	Merlot, Cabernet Sauvignon, Carmenere, Sauvignon Blanc
Austria	0	1	2	Gruner Veltliner, Grauburgunder, Neuburger, Pinot Blanc
South Africa	1	1	0	Merlot, Chenin Blanc
New Zealand	0	1	0	Gewurztraminer
Ukraine	0	1	0	Chardonnay
Argentina	0	1	0	Chardonnay
Czech Republic	0	0	1	Svatovavrinecke
Greece	0	2	0	Roditis
UK	0	0	1	-
Armenia	0	1	0	Kangun
**Total**	**79**	**75**	**26**	

**Table 4:** ICP-ToF-MS (OptiMass 8000, by GBC Melbourne, Australia,) and ICP-OES (Thermo Scientific, ICAP 7000 series, MA, USA) parameters and measurement conditions.

Parameter and Accessories	ICP-TOF-MS	ICP-OES
Number of replicates	3	3
Carrier gas	Argon	Argon
Plasma gas flow rate [L·min^−1^]	10	12
Auxiliary gas flow rate [L·min^−1^]	0.9	0.5
Nebulization gas flow rate [L·min^−1^]	0.76	0.5
Torch	Quartz	Quartz
Nebulizer	Concentric quartz	Concentric quartz
Generator power [W]	1330	1150
Internal standard	In	In
Plasma orientation	Axial	Axial or Radial

**Table 5:** Basic statistics for determined elements for all wine samples (*n* = 180) [µg/L].

Element	N	Mean	Median	Min	Max	Element	N	Mean	Median	Min	Max
Li	180	85.42	21.39	<LOD	1325	Rb	180	20,129	658.6	5.650	417,452
Be	180	0.054	0.021	<LOD	0.343	Mo	180	2379	6.210	<LOD	31,203
B	180	16,081	17,149	<LOD	63,969	Ag	180	9.770	3.340	<LOD	51.80
Cr	180	69.12	55.27	<LOD	534.8	Cd	180	2.744	<LOD	<LOD	31.04
Mn	180	910.1	804.1	35.37	3292	Te	180	0.186	0.009	<LOD	3.913
Fe	180	1569	1224	154.9	10,250	Ba	180	125.9	113.4	23.45	476.0
Co	180	14.41	7.410	0.080	59.20	Pb	180	50.28	38.20	<LOD	597.6
Cu	180	106.2	53.78	4.930	1071	Bi	180	12.40	1.000	0.003	78.00
Zn	180	250.4	117.8	<LOD	4046	U	180	0.408	0.269	<LOD	2.3457
Sr	180	605.2	438.2	95.99	3033	Mg	180	85,761	70,855	13,169	611,570
Sn	180	17.83	<LOD	<LOD	331.5	Ca	180	84,780	66,238	23,113	839,116
Sb	180	1.469	0.368	0.006	69.95	K	180	156,652	84,281	480.9	1,046,788
Ti	180	107.9	96.46	6.222	860.2	Tl	180	0.721	0.360	<LOD	1.221
Ni	180	40.59	35.69	<LOD	355.5						

**Table 6:** Basic statistical parameters of the pH results of all tested wines with a distinction between wine types.

Type of Wine	N	Mean	Median	Minimum	Maximum	St. Dev.	*p*-Value
**R**	79	3.33	3.30	2.53	4.60	0.32	0.03
**W**	75	3.01	3.02	2.42	3.69	0.30	0.64
**Ro**	26	2.89	2.88	2.42	3.48	0.25	0.86

**Table 7:** The mean concentration and median value of each metal content in white, red, and rose wines [µg/L].

Type of Wine	N		Li	Be	B	Cr	Mn	Fe	Ni	Co	Cu
Red	79	Mean	63.90	0.051	17,976	76.38	1172	1454	37.10	13.96	123.7
		Median	22.75	0.022	19,708	66.66	1049	1110	32.67	7.530	87.93
White	75	Mean	113.3	0.058	14,769	59.41	741.0	1530	41.86	16.53	84.91
		Median	17.84	0.019	16,189	45.33	715.8	1206	38.41	9.420	45.34
Rose	26	Mean	72.34	0.054	14,106	75.06	601.9	2031	47.51	9.660	114.3
		Median	18.14	0.021	16,012	43.61	443.4	1973	34.99	4.620	54.05
**Type of Wine**	**N**		**Rb**	**Mo**	**Ag**	**Cd**	**Te**	**Ba**	**Zn**	**Pb**	**Bi**
Red	79	Mean	10,993	3089	9.581	8.456	0.142	154.3	259.7	53.91	12.37
		Median	910.1	5.930	3.380	0.859	0.007	134.5	225.6	38.43	1.040
White	75	Mean	32,354	2256	11.01	9.416	0.263	104.9	259.7	41.98	14.19
		Median	590.8	6.250	4.107	4.200	0.012	93.35	96.73	38.02	1.950
Rose	26	Mean	12,623	579.9	6.751	5.856	0.129	100.4	195.6	63.24	7.299
		Median	432.6	7.050	2.541	0.403	<LOD	100.0	91.99	29.25	0.405
**Type of Wine**	**N**		**Ca**	**Mg**	**Ti**	**K**	**Sr**	**Sn**	**Sb**	**U**	**Tl**
Red	79	Mean	41,882	57,107	69.85	72,176	779.7	3.700	1.060	0.290	0.526
		Median	46,004	56,782	62.55	68,675	697.8	<LOD	<LOD	0.190	0.128
White	75	Mean	71,251	75,726	146.1	276,762	470.9	28.1	1.900	0.400	0.698
		Median	61,961	65,068	131.2	258,825	363.4	<LOD	0.500	0.300	0.269
Rose	26	Mean	51,700	72,244	113.8	66,799	462.2	31.03	1.400	0.690	0.822
		Median	37,582	71,803	96.46	62,544	361.1	<LOD	1.020	0.370	0.390

**Table 8:** Information on factor loadings.

Variables	F1	F2	F3	F4	F5	F6	F7	F8	F9	F10	F11
Ca	0.19	0.07	0.65	−0.53	0.07	−0.02	0.16	0.00	0.00	0.06	−0.01
Mg	0.19	−0.07	0.46	−0.69	0.19	0.01	0.24	−0.09	−0.09	0.14	−0.16
Ti	−0.02	0.12	0.38	−0.33	−0.46	0.39	−0.06	−0.08	0.04	0.10	−0.12
K	−0.01	0.20	−0.10	−0.25	−0.34	0.61	0.05	0.22	0.18	−0.28	0.06
Sr	0.05	−0.32	−0.45	−0.50	0.20	−0.23	−0.10	−0.02	−0.13	−0.04	−0.25
Sn	−0.13	−0.24	0.61	0.04	0.35	−0.04	0.14	−0.04	0.01	−0.15	0.17
Sb	0.11	0.07	0.13	0.05	0.05	0.00	−0.46	−0.34	0.68	0.30	−0.14
U	−0.14	−0.09	0.22	−0.20	−0.23	−0.12	−0.56	−0.19	−0.36	−0.40	0.00
Li	−0.62	0.55	−0.17	−0.29	−0.03	0.01	−0.08	−0.08	−0.04	−0.10	0.05
Be	−0.49	−0.71	0.17	0.20	−0.02	0.20	−0.06	−0.03	−0.11	0.00	−0.09
B	0.25	−0.67	−0.20	−0.06	−0.26	0.22	0.07	−0.23	−0.09	0.04	−0.15
Cr	−0.29	−0.08	−0.08	0.10	−0.53	−0.51	0.35	−0.08	0.09	0.01	−0.17
Mn	−0.06	−0.46	−0.51	−0.47	0.08	−0.16	−0.10	0.11	0.11	−0.12	−0.09
Fe	−0.17	−0.16	0.29	0.00	−0.53	−0.25	−0.32	0.06	−0.09	0.12	0.18
Ni	−0.15	−0.06	0.25	−0.07	−0.40	−0.57	0.18	0.20	0.30	−0.22	0.06
Co	−0.96	−0.06	0.03	0.03	0.03	0.03	0.05	0.03	0.03	0.02	−0.06
Cu	−0.08	−0.21	−0.16	−0.26	−0.21	0.01	−0.12	0.43	−0.17	0.62	0.25
Zn	−0.11	−0.59	−0.16	−0.13	−0.19	0.35	0.18	0.11	0.32	−0.18	0.06
Rb	−0.45	0.49	−0.17	−0.20	0.00	0.11	0.03	−0.06	0.02	−0.09	0.36
Mo	−0.58	0.52	−0.21	−0.20	0.10	−0.13	0.09	0.04	0.02	0.10	−0.13
Ag	−0.94	−0.17	0.13	0.08	0.09	0.06	0.00	0.02	−0.01	0.03	−0.05
Cd	−0.89	−0.35	0.15	0.13	0.09	0.05	−0.01	−0.01	−0.01	0.03	−0.02
Te	−0.62	0.43	−0.09	−0.20	−0.09	0.01	−0.10	−0.04	0.05	−0.01	−0.20
Ba	−0.21	−0.47	−0.05	−0.34	0.33	−0.21	−0.14	−0.03	0.28	−0.08	0.39
Tl	−0.98	−0.06	0.06	0.05	0.07	0.07	0.02	0.02	−0.01	0.05	−0.05
Pb	−0.19	−0.09	−0.30	−0.11	−0.21	0.02	0.28	−0.70	−0.11	0.15	0.32
Bi	−0.97	−0.05	0.05	0.04	0.06	0.06	0.02	0.02	0.00	0.07	−0.05
**% of Variance**	23.9	11.9	8.2	7.2	6.1	5.8	4.0	3.8	3.8	3.5	2.9
**% of Vumulative**	23.9	35.8	44.0	51.2	57.3	63.2	67.2	71.0	74.8	78.3	81.2

## Data Availability

Not applicable.

## Supplementary Materials

The following are available online: Figures S1–S26, Tables S1–S25.

## Sample Availability

Samples of the compounds are available from the authors.
