# Supplementary material for: Multi-Elemental Analysis of Wine Samples in Relation to Their Type, Origin, and Grape Variety
Source: Molecules. 2021 Jan 4;26(1):214. doi: 10.3390/molecules26010214 (PMC7795369; doi:10.3390/molecules26010214)
Supplement: Supplementary file 1 [file molecules-26-00214-s001.pdf]

Multi-Elemental Analysis of Wine Samples in Relation to Their Type, Origin, and Grape Variety

Magdalena Gajek *, Aleksandra Pawlaczyk and Malgorzata I. Szynkowska-Jozwik

Faculty of Chemistry, Institute of General and Ecological Chemistry, Lodz University of Technology, Zeromskiego 116, 90-924 Lodz, Poland; aleksandra.pawlaczyk@p.lodz.pl (A.P.); malgorzata.szynkowska@p.lodz.pl (M.I.S.-J.)

* Correspondence: magdalena.gajek@edu.p.lodz.pl; Tel.: +48-42-631-30-95

Table S1. A literature review on the content of metals in wines.

**Samples**	**Elements**	**Technique**	**Preparation**	**Conclusions**	**Ref.**
60 white wines from Kosovo	Zn, Fe, Cu, Mn, Hg, As	FAAS<br>HGAAS	Mineralization 100 mL sample + 1 mL HNO_3 (con.)	Max. content of metals:<br>Zn 0.67 mg/L; Fe 2.03 mg/L; Cu 0.84 mg/L; Mn 1.17 mg/L; Hg 6.40 μg/L; As 6.56 μg/L	[2]
13 samples of different wine brands from Croatia: 5 white, 5 red, 1 rosé and 2 fruit wines	Pb, Cd, Cu, Zn	FAAS<br>ZGFAAS<br>FTSCP	Filtering + dilution + 0.1M HCl	Mean content of metals [μg/L]:<br>Pb 9.5; Cd 0.13; Cu 156; Zn 640	[27]
17 homemade fruit wine from Poland	K, Ca, Fe, Zn, Cd, Mg, Pb, Sn, Hg	AES<br>AAS<br>CVAAS<br>GFAAS	Mineralization 10 mL sample + 5 mL HNO_3 + 5 mL H_2O_2	Max. content of metals [μg/L]:<br>Pb 166.3; Zn 316; Cd 18.4; Hg 0.437; Fe 969	[25]
60 wines from Romania and Moldova including 26 red wines (18 different grape varieties)	Ni, Ag, Cr, Sr, Zn, Cu, Rb, Mn, Pb, Co, V	ICP-MS	Mineralization 2,5 mL sample + 2,5 mL HNO_3 (con.)	Max. content of metals [μg/L]:<br>Rb 890; Mn 865; Cu 2594.79; Zn 433,95; Ni 324,73	[6]
4 different brands of wine purchased in Polish market: 1 white and 3 red wine samples (4 different grape varieties)	Al, B, Ba Ca, Cu, Fe, K, Li, Mg, Mn, Na, P, Se, Sr, Zn	ICP-OES	1. Dilution (1:10)<br>2. Microwave-Assisted Digestion (2 mL of sample + 8 mL HNO3(con.))<br>3. Water Bath-Assisted Digestion (2 mL of sample + 8 mL HNO3(con.) + heating)	Range of determined elements (sample prepare in three ways) [mg/L]:<br>Li 0.057 - 0.091<br>Ba 0.119 – 0.285<br>Mn 0.57 – 1.62<br>B 2.44 – 5.26	[23]
44 Polish wines coming from directly 9 Polish vineyards (24	30 elements, e.g.: Ag, Al, Ba, Bi, Hg, Mn, Ni, Ti, Tl, V	ICP-MS<br>ICP-OES	Dilution of the sample (1:10)	Mean content of metals in white wines [μg/L]:<br>Ag 1.4; Al 710; Ba 310; Bi 49; Hg 0.4; Mn 318; Ni 64; Ti 33; Tl 0.87; V 8.2;	[5]

white and 20 red wines)					
10 red wines from Croatia gained directly for vineyards (10 different producers)	Cu, Fe, Zn, Pb	AAS	Mineralization 20 mL sample + Dissolution of the residue in 10 mL 10% HNO_3	Range of determined elements [mg/L]:<br>Cu 0.24 – 1.12<br>Fe 0.81 – 6.20<br>Zn 0.27 – 2.43<br>Pb 0.11 – 0.34	[28]
1 sample of wine from Croatia	V, Cr, Mn, Fe, Ni, Cu, Zn, As, Pb	XRF	10 mL of samples + 30% H_2O_2 + evaporation + 0,5M HNO_3	Max. content of metals [μg/L]:<br>V 1; Cr 3; Mn 5; Fe 61; Ni 6; Cu 6; Zn 59; Pb 30;	[30]
4 sample of different brand of red wines from Madeira (Portugal)	Ti, Cr, Mn, Fe, Ni, Cu, Zn, Rb, Sr, Pb	XRF	Mineralization 2 mL sample + 2 mL HNO_3(con.)	Max. content of metals [mg/L]:<br>Ti 0.13; Cr ≤ 0.02; Mn 2.05; Fe 5.3; Ni 0.06; Cu 1.73; Zn 2.63; Rb 1.24; Sr 1.01; Pb ≤ 0.08;	[31]
60 samples of red (50) and white (10) wines purchased in Korea coming from various countries including France and Australia	Pb, Cd	GFAAS	Mineralization 5 mL sample + 5 mL HNO_3(con.)	Mean content of metals [μg/L]:<br>Pb 29; Cd 0.5	[26]
153 samples of wine (red, white and rosé) wines from the Canary Islands (Spain)	39 trace and ultratrace elements e.g.: La, U, Te, Er	ICP-MS	Dilution of the sample (1:10)	Range of rare earth elements: 0.01 – 5.82 μg/L<br>Range of other elements: 0.011 – 5327 μg/L<br>Te 0.1 – 0.43 μg/L	[24]
10 wines (5 red and 5 white) from Austria	Be	AAS	Wet-ashed method	Range of Be [μg/L]: <LOD - 0.15	[29]
40 wines from three producing regions in South Africa (17 red and 23 white wines)	40 elements e.g.: Li, B, W, Ba, U, Rb, Se	ICP - MS	1. Dilution (1:1) with 0.14 M HNO_3<br>2. Mineralization 1.5 mL sample + 150 μL HNO_3(con.) + 1.5 mL H_2O_2 (for rare earth elements)	Range of B [mg/L]: 2.55 – 3.5	[22]
20 samples from 4 different brands of Ethiopian wines (2 white and 2 red wines)	13 elements e.g.: K, Na, Ca, Mg, Fe, Zn, Mn	FAAS	Digestion 100 mL sample + HNO_3(con.) + H_2O_2(30%)	Range of some elements [mg/L]:<br>K 694–767<br>Ca 28.4–37.1<br>Mg 58.1–79.2	[19]

31 wines from three different regions in Argentina	11 elements e.g.: Ca, Mn, Fe, Zn, Cu	FAAS, FAES	Dilution 1% HNO_3 in the range of 1 - 500 times	Median value for Ca for different regions of Argentina [mg/L]:<br>Córdoba – 50<br>La Rioja – 31<br>San Juan - 286	[3]
53 samples of red wine from Argentina (13), Brazil (15), Chile (13) and Uruguay (12)	45 elements e.g.: Ti, Be, Bi, Ca, Tl, Ag, U, Sn, Sr	ICP-MS, ICP-OES	1 ml sample + 3 ml HNO_3 (heating) + dilution to 25 mL	Means values for Ti and U [µg/L]:<br>Argentina Ti – 137; U – 1.2<br>Brazil Ti – 126; U – 0.2<br>Chile Ti - 143; U – 0.4<br>Uruguay Ti - 143; U – 0.7	[21]

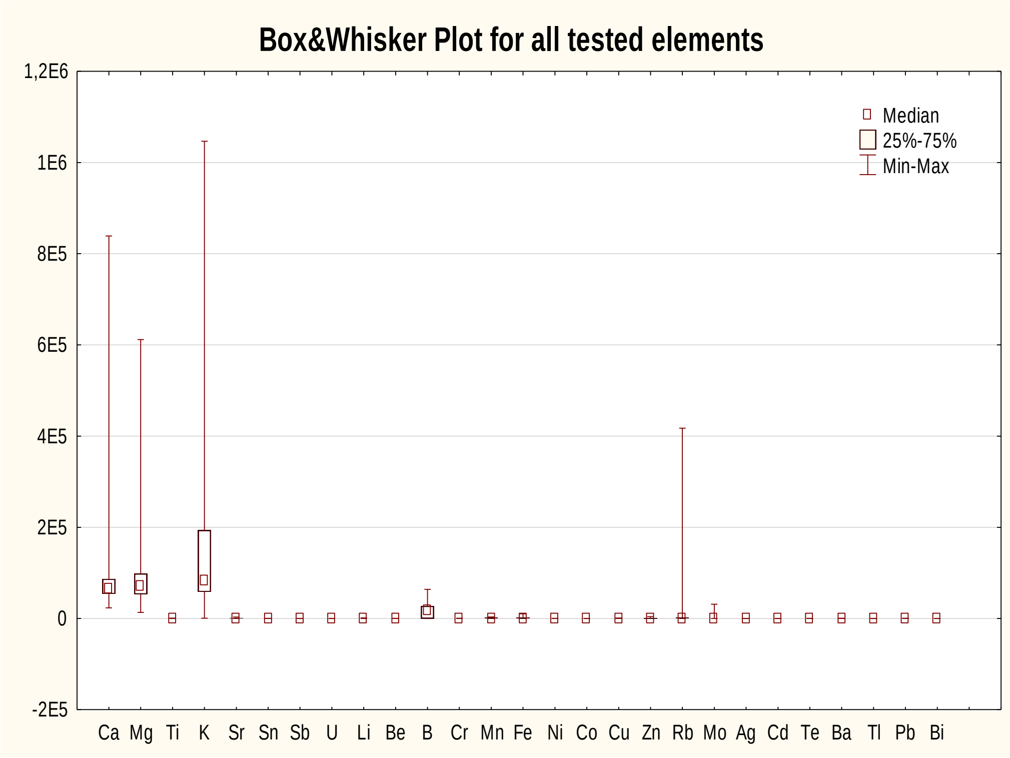

Figure S1. Box&Whisker Plot for content of all determined elements in 180 samples of wine [µg/L].

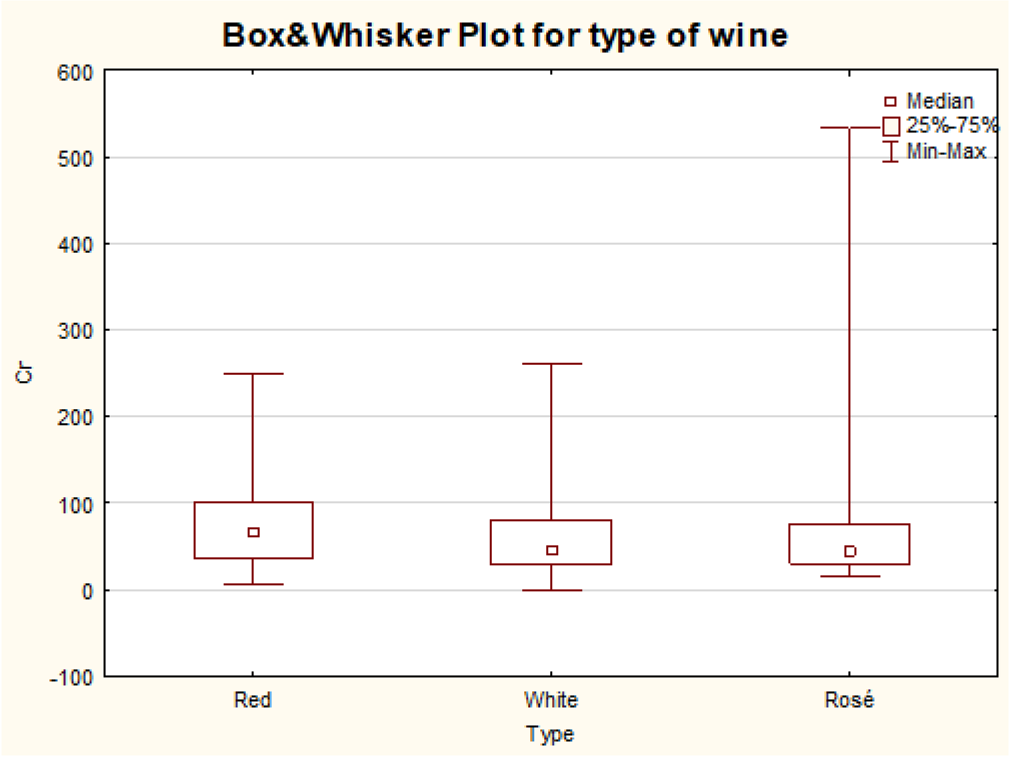

Figure S2. Box&Whisker Plot for content of Cr for obtained results of 180 samples of wine with division according to type [μg/L].

Table S2. Basic statistics of Cr for all wine samples with division according to type (n = 180) [μg/L].

Color	N	Mean	Median	Min	Max	Std. Dev.
Red	79	76.38	66.66	5.130	249.1	49.03
White	75	59.41	45.33	<LOD	262.0	45.15
Rosé	26	75.06	43.61	14.62	534.8	106.2

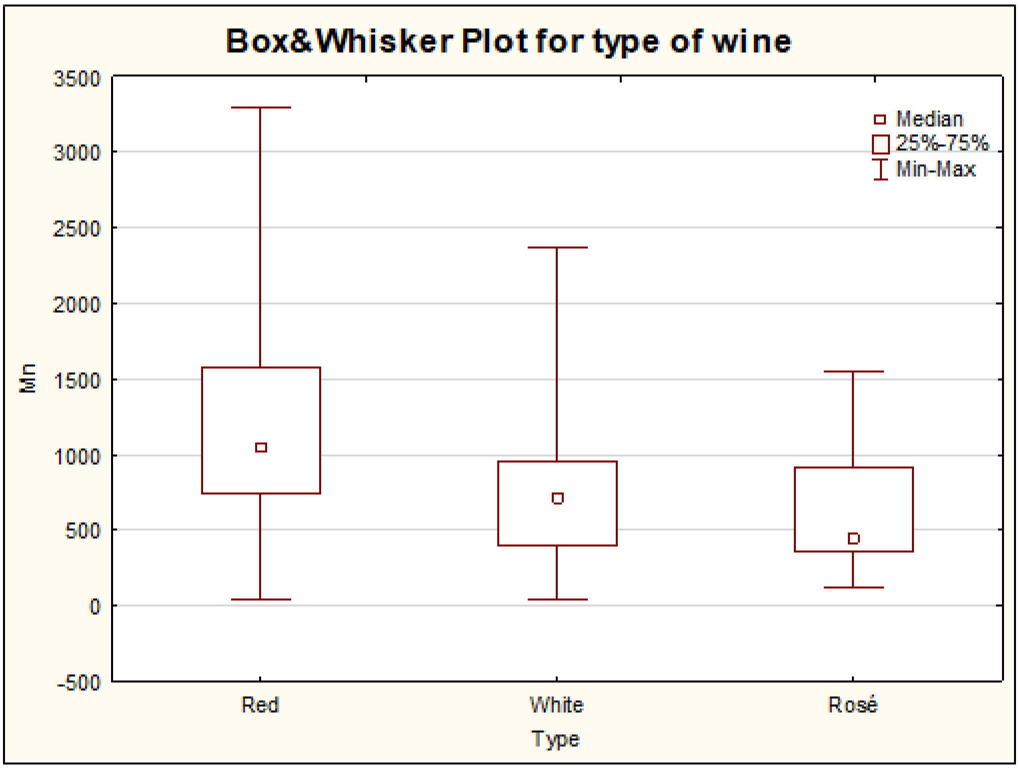

Figure S3. Box&Whisker Plot for content of Mn for obtained results of 180 samples of wine with division according to type [μg/L].

Table S3. Basic statistics of Mn for all wine samples with division according to type (n = 180) [μg/L].

Color	N	Mean	Median	Min	Max	Std. Dev.
Red	79	1172	1050	35.37	3293	690.9
White	75	741.0	715.8	40.01	2376	448.4
Rosé	26	601.9	443.4	124.7	1544	352.7

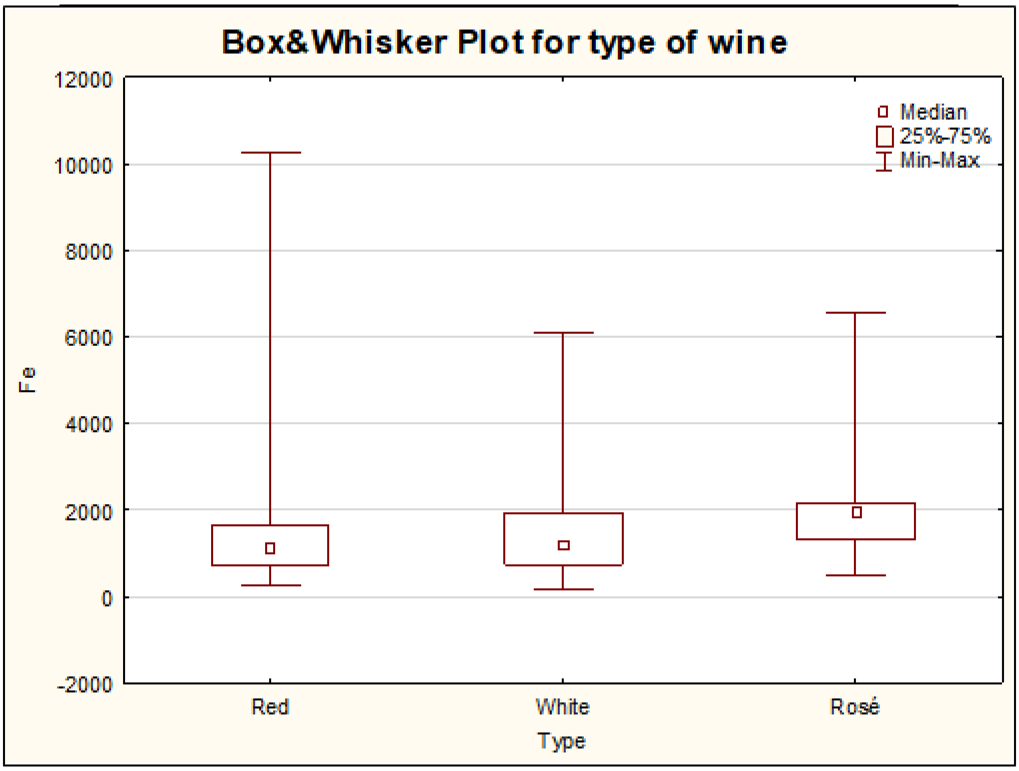

Figure S4. Box&Whisker Plot for content of Fe for obtained results of 180 samples of wine with division according to type [μg/L].

Table S4. Basic statistics of Fe for all wine samples with division according to type (n = 180) [μg/L].

Color	N	Mean	Median	Min	Max	Std. Dev.
Red	79	1454	1111	281.9	10250	1323
White	75	1530	1207	154.9	6093	1126
Rosé	26	2031	1973	483.8	6557	1252

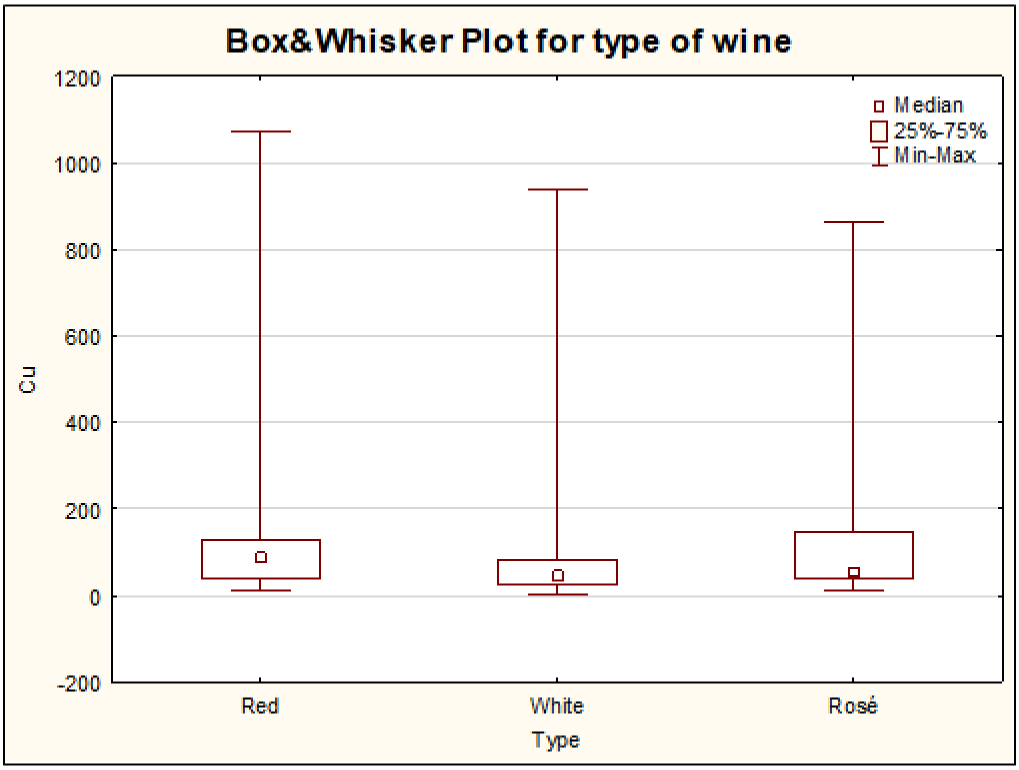

Figure S5. Box&Whisker Plot for content of Cu for obtained results of 180 samples of wine with division according to type [μg/L].

Table S5. Basic statistics of Cu for all wine samples with division according to type (n = 180) [μg/L].

Color	N	Mean	Median	Min	Max	Std. Dev.
Red	79	123.7	87.93	10.49	1072	148.4
White	75	84.91	45.34	4.930	939.1	135.3
Rosé	26	114.3	54.05	14.04	865.0	168.1

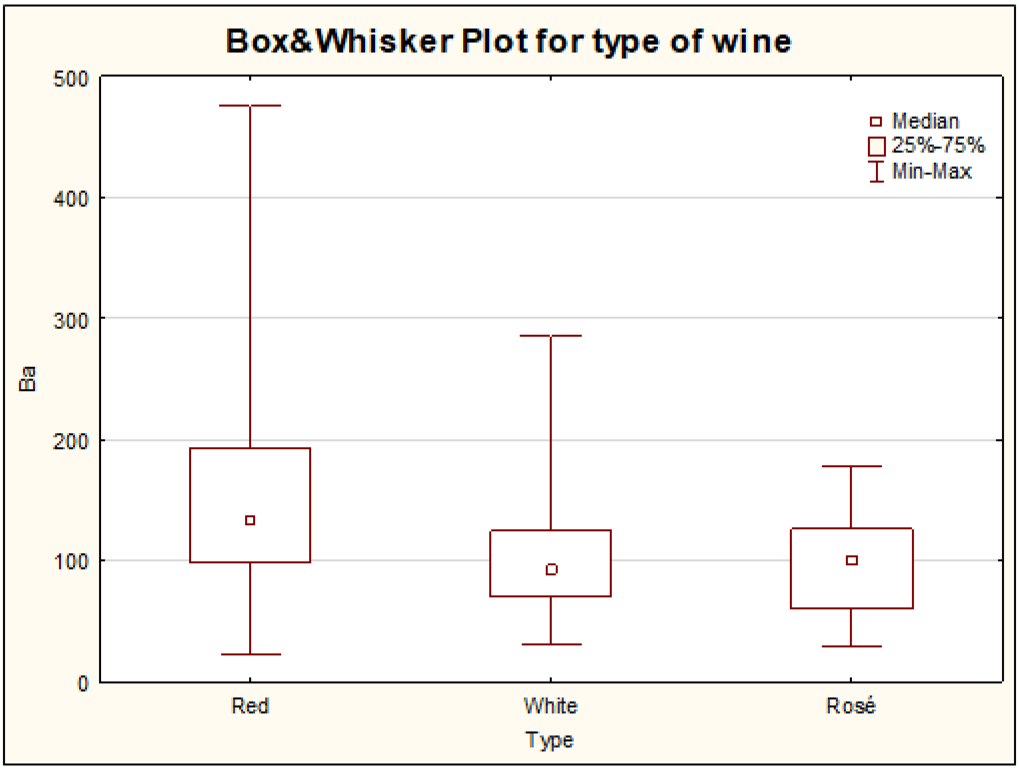

Figure S6. Box&Whisker Plot for content of Ba for obtained results of 180 samples of wine with division according to type [μg/L].

Table S6. Basic statistics of Ba for all wine samples with division according to type (n = 180) [μg/L].

Color	N	Mean	Median	Min	Max	Std. Dev.
Red	79	154.3	134.5	23.45	476.0	80.96
White	75	104.9	93.35	30.66	286.0	49.36
Rosé	26	100.4	100.0	29.37	179.1	44.92

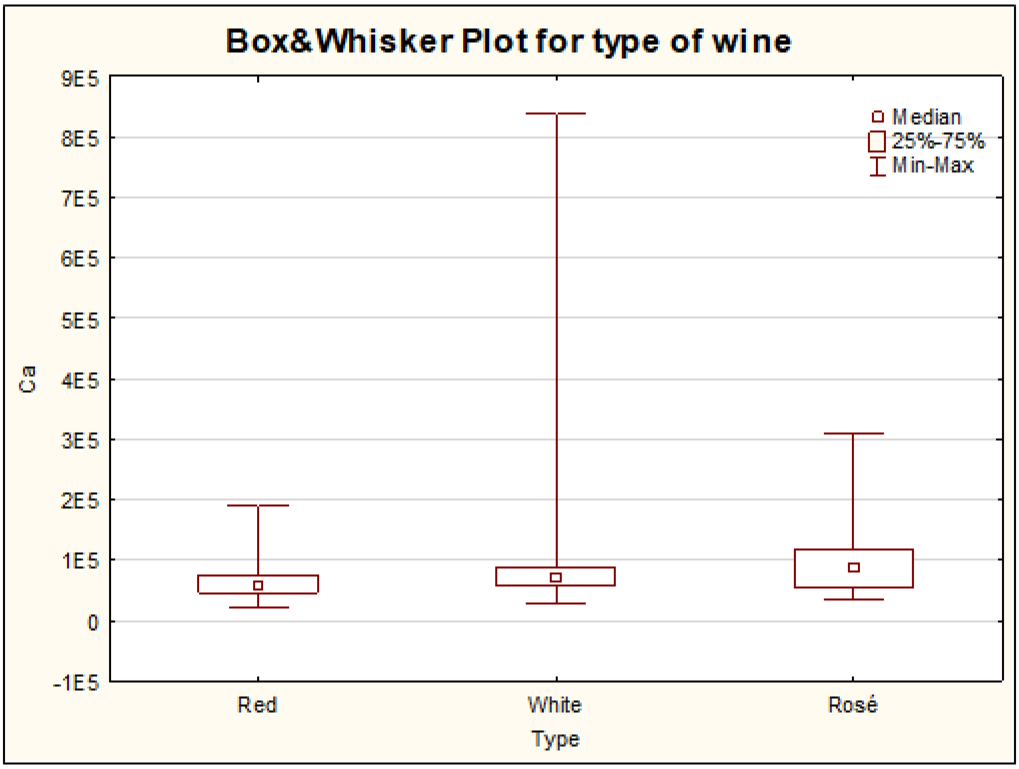

Figure S7. Box&Whisker Plot for content of Ca for obtained results of 180 samples of wine with division according to type [µg/L].

Table S7. Basic statistics of Ca for all wine samples with division according to type (n = 180) [µg/L].

Color	N	Mean	Median	Min	Max	Std. Dev.
Red	79	65669	59537	23113	190352	27654
White	75	101238	70552	30175	839115	109050
Rosé	26	95370	87996	33876	311317	56394

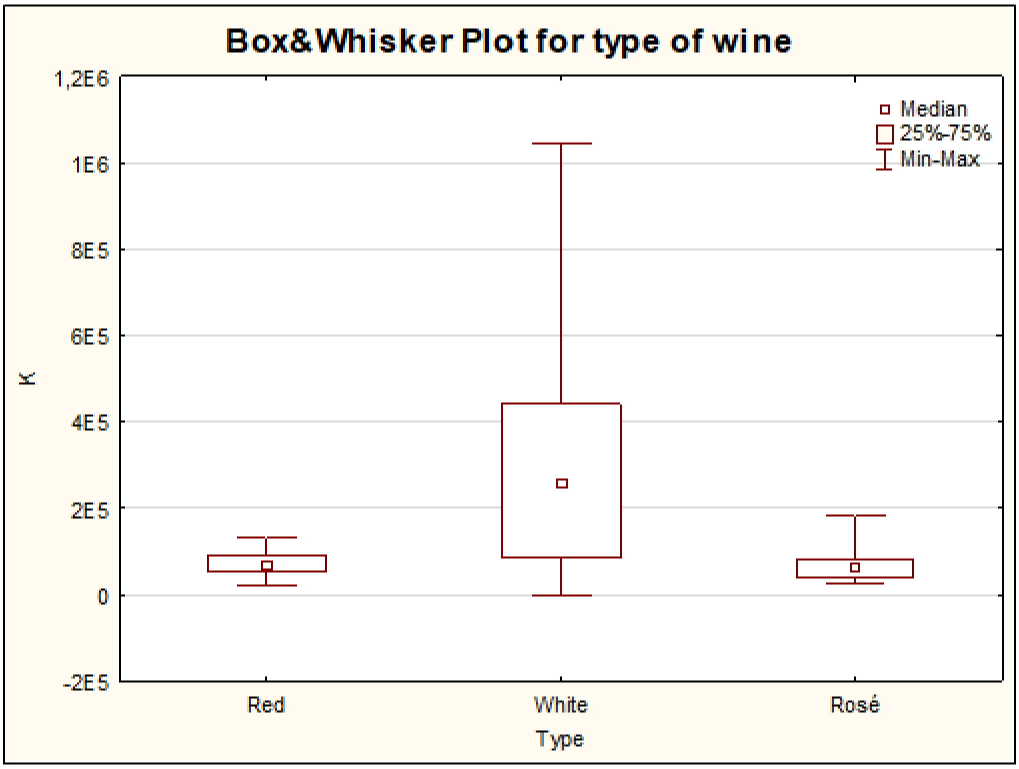

Figure S8. Box&Whisker Plot for content of K for obtained results of 180 samples of wine with division according to type [μg/L].

Table S8. Basic statistics of K for all wine samples with division according to type (n = 180) [μg/L].

Color	N	Mean	Median	Min	Max	Std. Dev.
Red	79	72176	68676	21475	132932	25811
White	75	276782	258826	480.9	1046788	210305
Rosé	26	66799	62545	28422	183589	33994

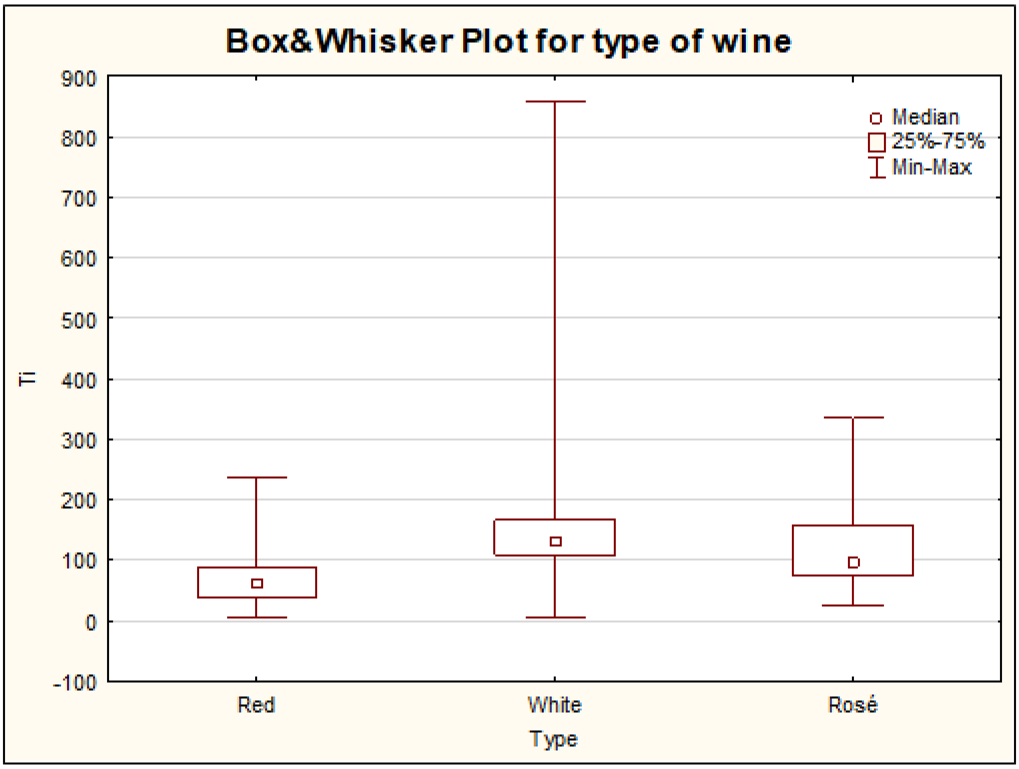

Figure S9. Box&Whisker Plot for content of Ti for obtained results of 180 samples of wine with division according to type [μg/L].

Table S9. Basic statistics of Ti for all wine samples with division according to type (n = 180) [μg/L].

Color	N	Mean	Median	Min	Max	Std. Dev.
Red	79	69.85	62.55	6.360	236.0	43.84
White	75	146.1	131.2	6.222	860.2	102.0
Rosé	26	113.8	96.46	24.72	335.2	63.88

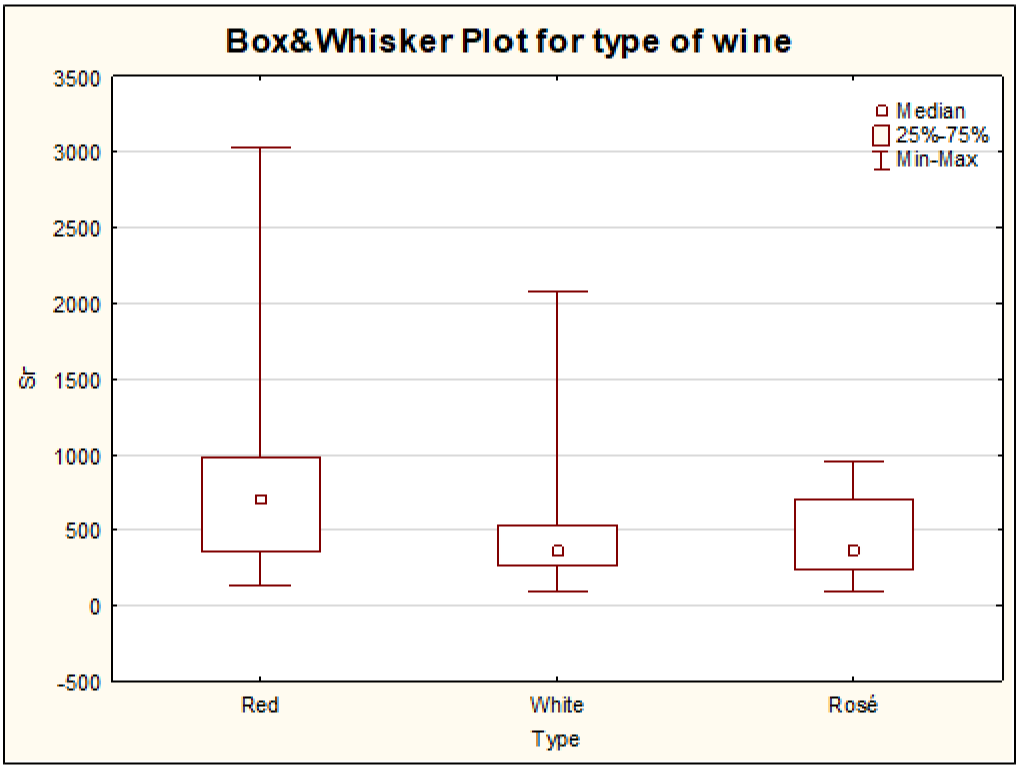

Figure S10. Box&Whisker Plot for content of Sr for obtained results of 180 samples of wine with division according to type [µg/L].

Table S10. Basic statistics of Sr for all wine samples with division according to type (n = 180) [µg/L].

Color	N	Mean	Median	Min	Max	Std. Dev.
Red	79	779.7	697.8	134.4	3034	559.3
White	75	470.9	363.4	98.54	2079	353.0
Rosé	26	462.2	361.1	95.99	951.8	282.2

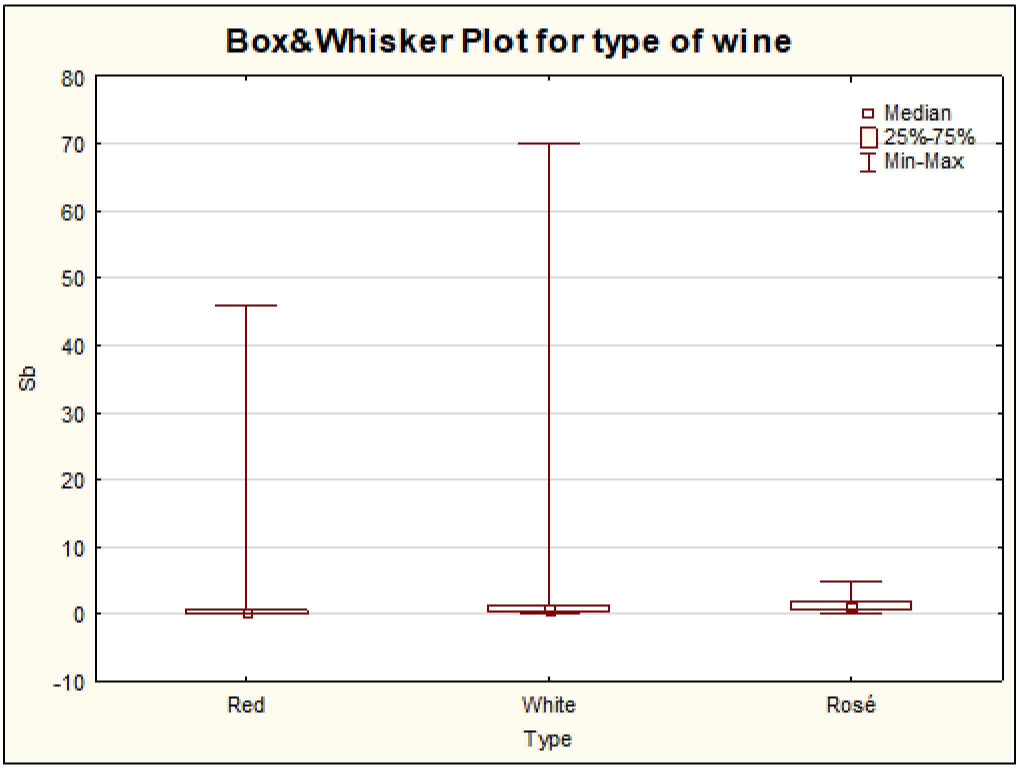

Figure S11. Box&Whisker Plot for content of Sb for obtained results of 180 samples of wine with division according to type [µg/L].

Table S11. Basic statistics of Sb for all wine samples with division according to type (n = 180) [µg/L].

Color	N	Mean	Median	Min	Max	Std. Dev.
Red	79	1.058	0.098	<LOD	45.91	5.279
White	75	1.928	0.481	<LOD	69.95	8.065
Rosé	26	1.398	1.023	<LOD	4.979	1.276

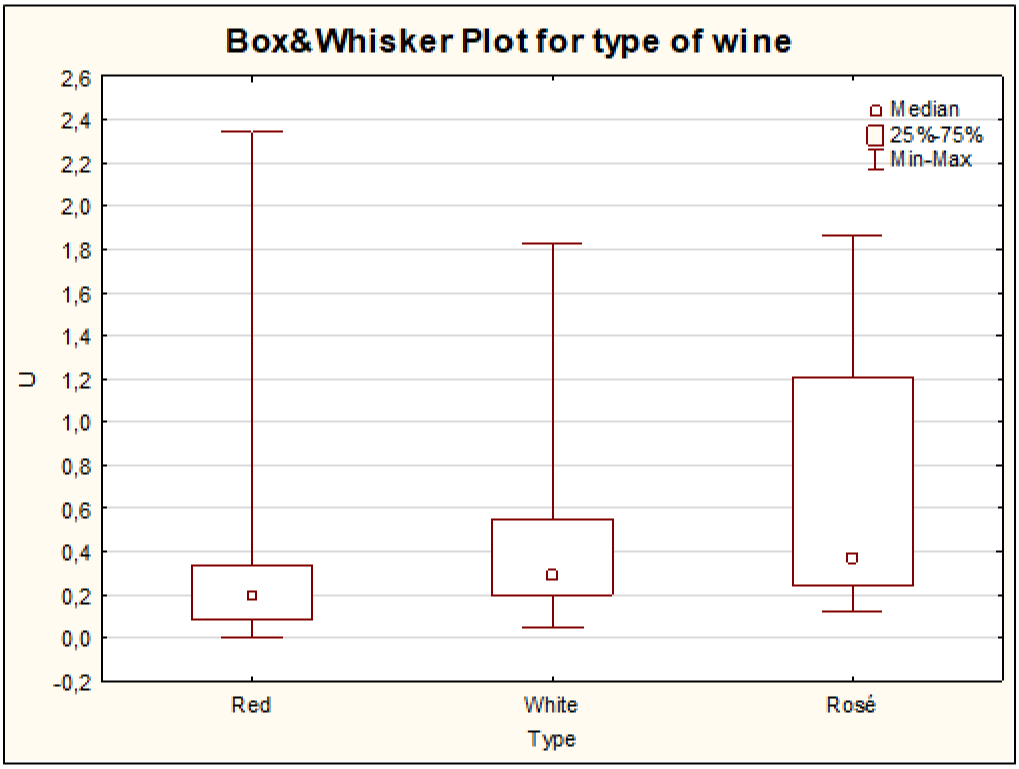

Figure S12. Box&Whisker Plot for content of U for obtained results of 180 samples of wine with division according to type [μg/L].

Table S12. Basic statistics of U for all wine samples with division according to type (n = 180) [μg/L].

Color	N	Mean	Median	Min	Max	Std. Dev.
Red	79	0.291	0.194	<LOD	2.345	0.390
White	75	0.434	0.297	0.048	1.829	0.375
Rosé	26	0.692	0.369	0.123	1.867	0.614

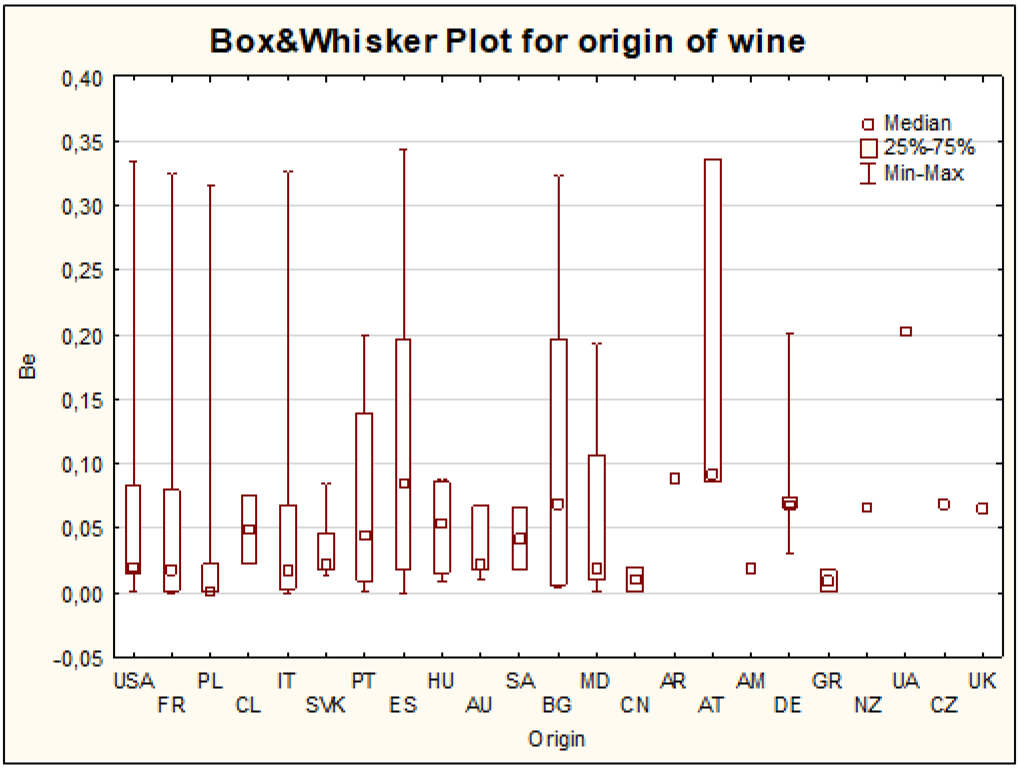

Figure S13. Box&Whisker Plot for content of Be for obtained results of 180 samples of wine with division according to country of origin [μg/L].

Table S13. Basic statistics of Be for all wine samples with division according to country of origin (n = 180) [μg/L].

Origin	N	Mean	Median	Min	Max	Std. Dev.
USA	18	0.076	0.020	0.001	0.334	0.104
FR	16	0.050	0.018	0.000	0.325	0.081
PL	42	0.026	<LOD	0.000	0.316	0.058
CL	2	0.049	0.049	0.022	0.076	0.038
IT	31	0.042	0.018	0.000	0.326	0.067
SVK	8	0.034	0.022	0.014	0.085	0.027
PT	8	0.073	0.045	0.001	0.199	0.082
ES	15	0.095	0.085	0.000	0.343	0.100
HU	4	0.051	0.053	0.009	0.088	0.041
AU	7	0.033	0.022	0.010	0.067	0.024
SA	2	0.042	0.042	0.018	0.066	0.034
BG	5	0.120	0.069	0.005	0.324	0.138
MD	3	0.058	0.019	0.001	0.193	0.091
CN	2	0.010	0.010	0.001	0.020	0.014
AR	1	0.089	0.089	0.089	0.089	-
AT	3	0.172	0.092	0.086	0.336	0.143
AM	1	0.019	0.019	0.019	0.019	-
DE	5	0.088	0.068	0.031	0.201	0.065
GR	2	0.009	0.009	0.000	0.018	0.012
NZ	1	0.067	0.067	0.067	0.067	-
UA	1	0.202	0.202	0.202	0.202	-

CZ	1	0.068	0.068	0.068	0.068	-
UK	1	0.065	0.065	0.065	0.065	-

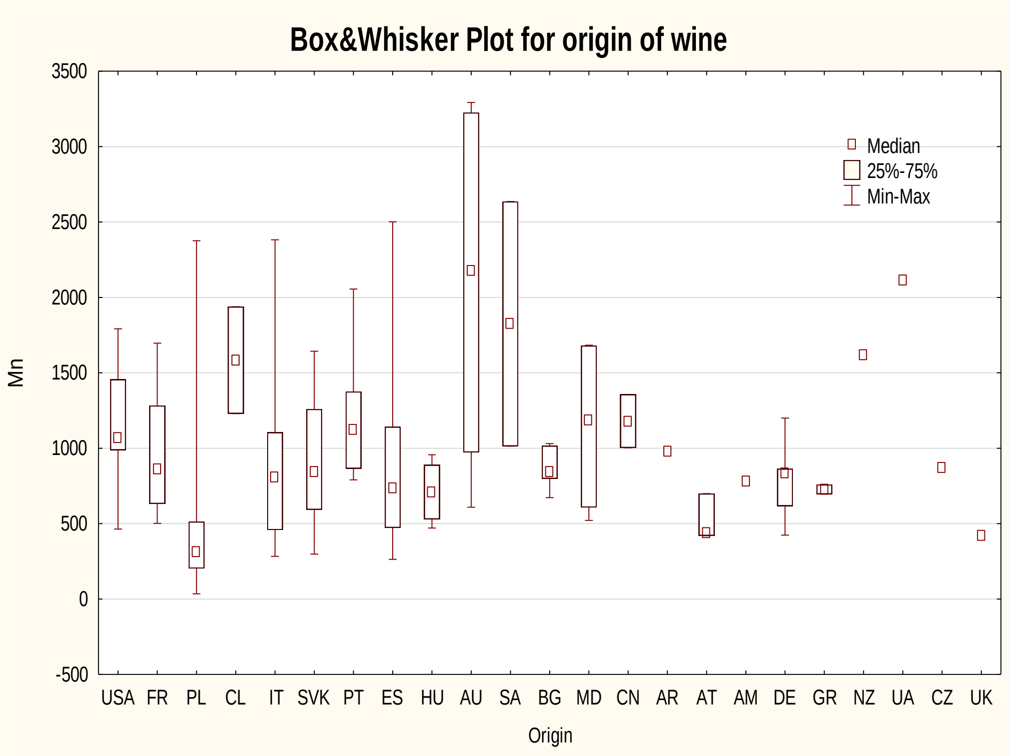

Figure S14. Box&Whisker Plot for content of Mn for obtained results of 180 samples of wine with division according to country of origin [μg/L].

Table S14. Basic statistics of Mn for all wine samples with division according to country of origin (n = 180) [μg/L].

Origin	N	Mean	Median	Min	Max	Std. Dev.
USA	18	1188	1069	464.3	1792	334.0
FR	16	962.2	862.6	501.9	1697	372.9
PL	42	452.5	314.9	35.37	2376	451.1
CL	2	1584	1584	1229	1938	501.3
IT	31	923.1	805.9	282.9	2383	524.5
SVK	8	917.5	847.2	298.1	1644	448.0
PT	8	1196	1118	790.9	2056	418.9
ES	15	876.8	732.6	262.8	2502	588.1
HU	4	709.7	705.4	471.2	956.9	220.0
AU	7	2097	2179	608.8	3293	1087
SA	2	1825	1825	1013	2636	1147
BG	5	872.9	846.8	672.4	1030	151.4
MD	3	1144	1185	521.9	1683	623.5
CN	2	1180	1180	1003	1357	250.4
AR	1	978.3	978.3	978.3	978.3	-
AT	3	520.3	441.3	420.5	698.9	155.1
AM	1	779.2	779.2	779.2	779.2	-
DE	5	787.6	833.0	424.0	1200	291.3

GR	2	727.2	727.2	695.2	759.1	45.20
NZ	1	1618	1618	1618	1618	-
UA	1	2112	2112	2112	2112	-
CZ	1	867.2	867.2	867.2	867.2	-
UK	1	421.4	421.4	421.4	421.4	-

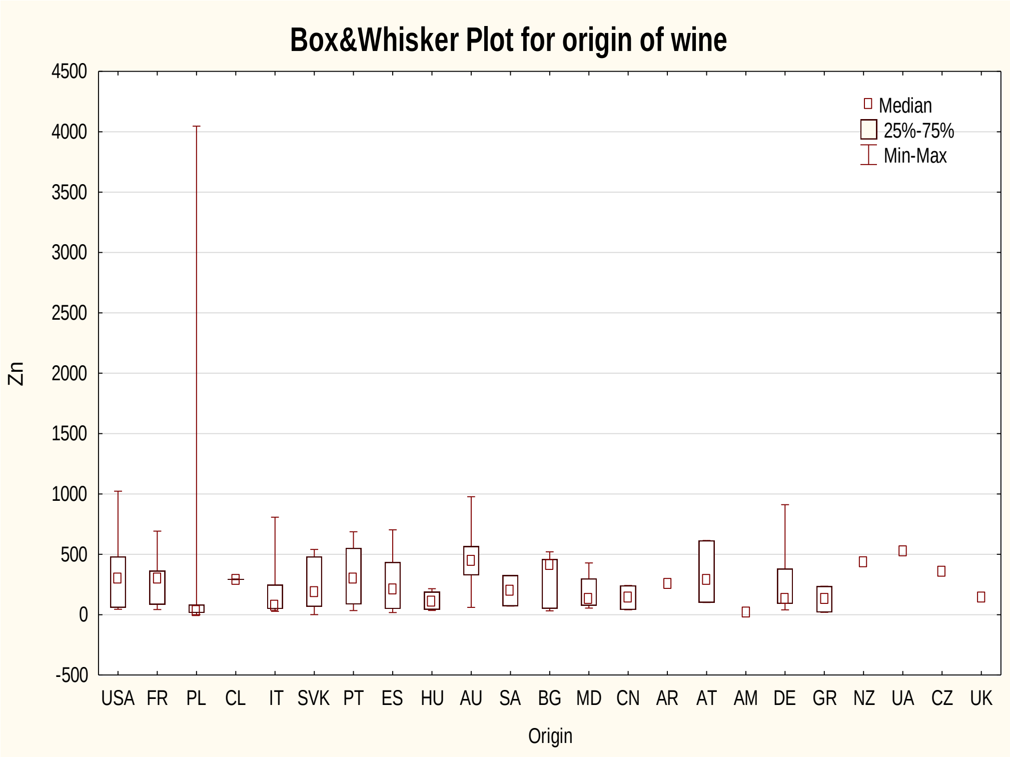

Figure S15. Box&Whisker Plot for content of Zn for obtained results of 180 samples of wine with division according to country of origin [μg/L].

Table S15. Basic statistics of Zn for all wine samples with division according to country of origin (n = 180) [μg/L].

Origin	N	Mean	Median	Min	Max	Std. Dev.
USA	18	331.1	296.2	44.84	1023	279.8
FR	16	251.6	297.7	42.40	692.1	184.2
PL	42	212.4	30.53	<LOD	4046	668.0
CL	2	292.9	292.9	290.0	295.8	4.101
IT	31	185.4	71.03	28.05	807.4	223.8
SVK	8	251.4	185.8	1.100	540.5	220.7
PT	8	324.6	299.6	34.26	687.0	256.5
ES	15	269.5	205.3	17.43	703.1	218.7
HU	4	116.4	108.0	34.95	214.9	87.36
AU	7	468.3	441.2	60.55	977.7	279.5
SA	2	198.7	198.7	71.22	326.2	180.3
BG	5	294.8	410.5	31.95	520.8	234.8
MD	3	187.4	133.6	54.14	428.4	167.7
CN	2	141.7	141.7	41.43	241.9	141.8
AR	1	257.5	257.5	257.5	257.5	-

AT	3	333.4	284.9	100.5	614.7	260.5
AM	1	17.40	17.40	17.40	17.40	-
DE	5	309.9	127.1	39.91	911.2	360.8
GR	2	127.8	127.8	19.58	236.1	153.1
NZ	1	436.0	436.0	436.0	436.0	-
UA	1	525.7	525.7	525.7	525.7	-
CZ	1	357.4	357.4	357.4	357.4	-
UK	1	137.4	137.4	137.4	137.4	-

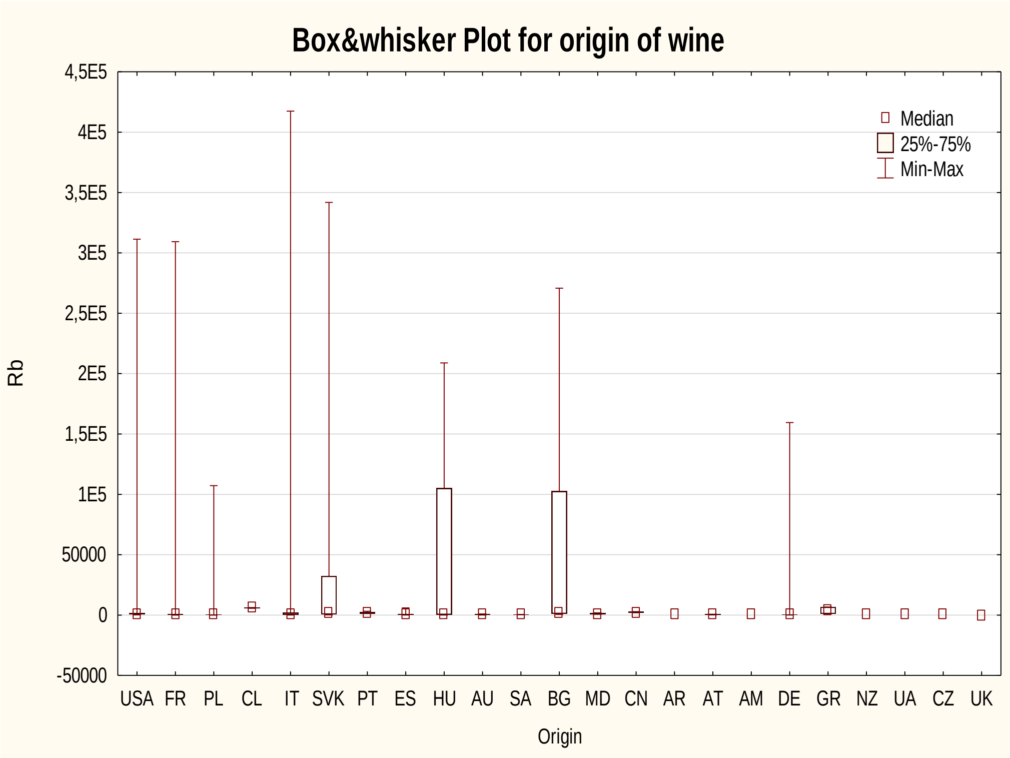

Figure S16. Box&Whisker Plot for content of Rb for obtained results of 180 samples of wine with division according to country of origin [μg/L].

Table S16. Basic statistics of Rb for all wine samples with division according to country of origin (n = 180) [μg/L].

Origin	N	Mean	Median	Min	Max	Std. Dev.
USA	18	18311	1249	140.4	311332	73130
FR	16	42211	537.5	32.18	309332	97923
PL	42	6164	329.5	5.650	107197	23193
CL	2	5956	5956	5661	6251	417.3
IT	31	36495	1132	35.79	417452	100941
SVK	8	51462	1865	131.4	341837	119222
PT	8	1894	1653	1294	3232	685.8
ES	15	940.7	623.1	40.15	6008	1455
HU	4	52675	842.9	79.45	208933	104173
AU	7	537.2	496.6	106.1	947.2	264.1
SA	2	441.1	441.1	362.1	520.1	111.7
BG	5	75313	1431	626.0	270782	117783

MD	3	1231	1162.1	749.8	1849	526.7
CN	2	2409	2409	1991	2827	591.0
AR	1	424.5	424.5	424.5	424.5	-
AT	3	499.7	629.0	202.5	667.7	258.1
AM	1	405.1	405.1	405.1	405.1	-
DE	5	32070	230.2	52.69	159500	71236
GR	2	3892	3892	1103	6681	3944
NZ	1	940.7	940.7	940.7	940.7	-
UA	1	621.4	621.4	621.4	621.4	-
CZ	1	1318	1318	1318	1318	-
UK	1	205.3	205.3	205.3	205.3	-

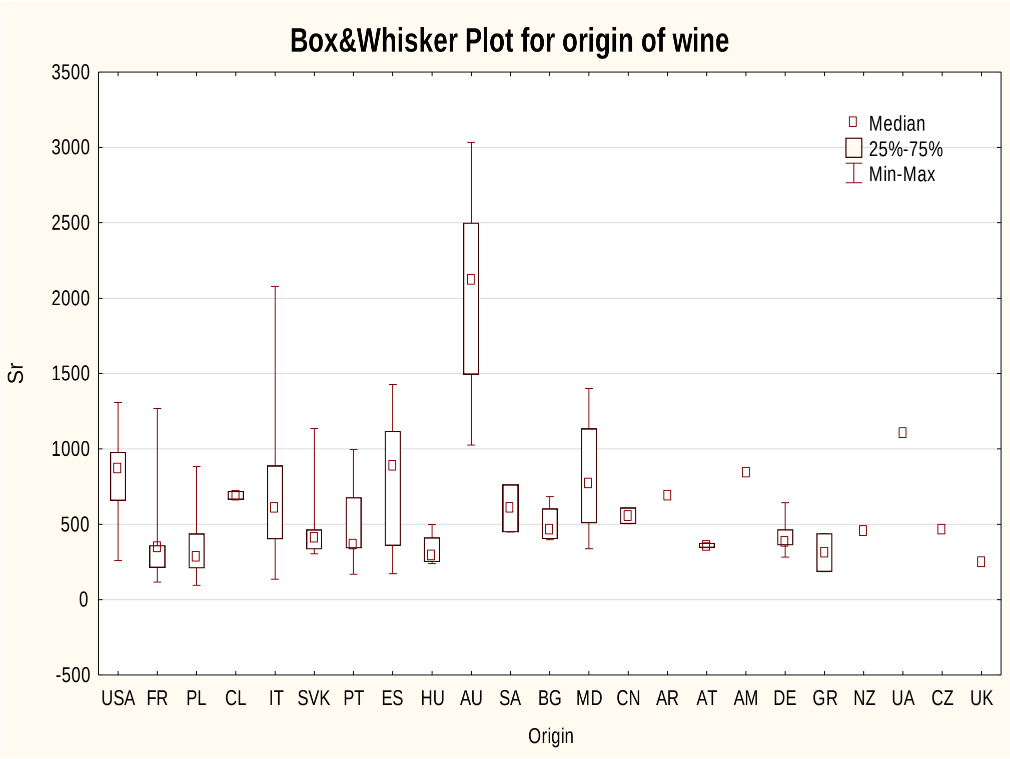

Figure S17. Box&Whisker Plot for content of Sr for obtained results of 180 samples of wine with division according to country of origin [μg/L].

Table S17. Basic statistics of Sr for all wine samples with division according to country of origin (n = 180) [μg/L].

Origin	N	Mean	Median	Min	Max	Std. Dev.
USA	18	798.8	874.3	259.6	1310	311.8
FR	16	384.4	343.6	116.3	1270	280.6
PL	42	336.9	288.5	96.0	883.8	193.0
CL	2	692.5	692.5	664.9	720.0	38.99
IT	31	701.1	613.0	136.0	2079	448.8
SVK	8	481.3	406.7	303.9	1136	271.7
PT	8	491.1	361.9	168.4	997.5	305.7
ES	15	770.2	890.6	171.9	1427	409.1
HU	4	331.4	293.6	239.2	499.0	117.1

AU	7	2094	2123	1025.8	3034	664.9
SA	2	605.3	605.3	447.9	762.8	222.7
BG	5	511.0	467.5	397.0	683.3	127.0
MD	3	822.1	774.2	337.7	1402	444.6
CN	2	557.2	557.2	504.3	610.1	74.82
AR	1	689.4	689.4	689.4	689.4	-
AT	3	361.0	360.4	345.6	376.9	15.66
AM	1	840.9	840.9	840.9	840.9	-
DE	5	427.3	385.3	282.1	642.9	136.9
GR	2	312.1	312.1	185.6	438.7	179.0
NZ	1	453.6	453.6	453.6	453.6	-
UA	1	1107	1107	1107	1107	-
CZ	1	463.4	463.4	463.4	463.4	-
UK	1	248.1	248.1	248.1	248.1	-

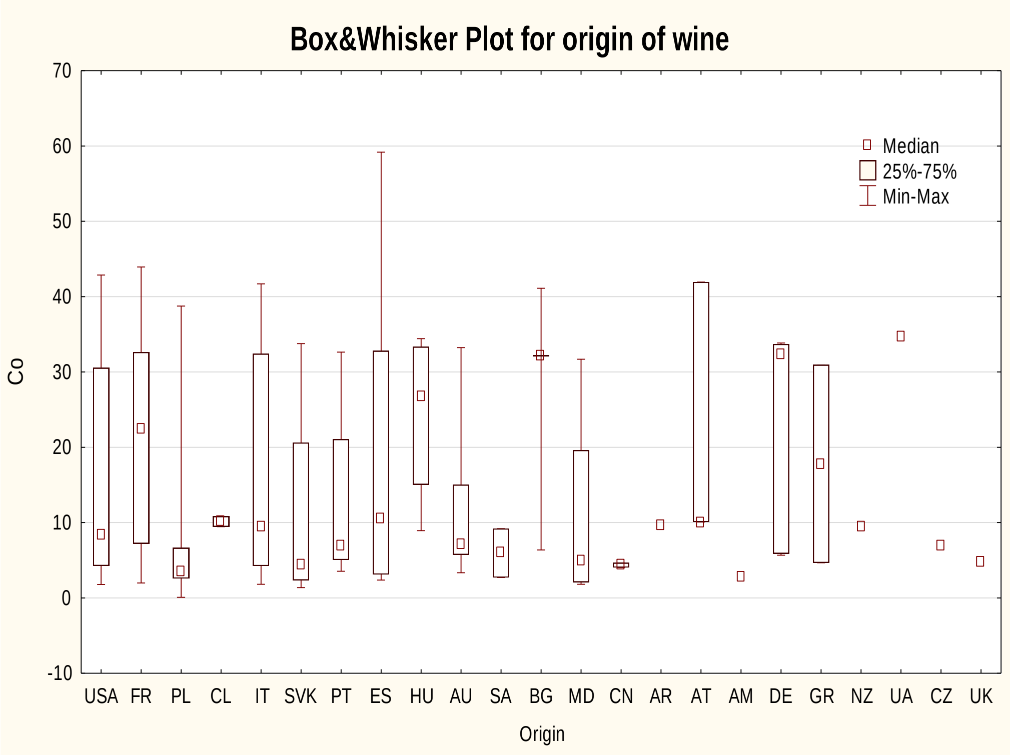

Figure S18. Box&Whisker Plot for content of Co for obtained results of 180 samples of wine with division according to country of origin [μg/L].

Table S18. Basic statistics of Co for all wine samples with division according to country of origin (n = 180) [μg/L].

Origin	N	Mean	Median	Min	Max	Std. Dev.
USA	18	14.64	8.352	1.780	42.87	14.34
FR	16	20.42	22.53	1.990	43.95	14.51
PL	42	8.377	3.621	0.080	38.75	10.90
CL	2	10.15	10.15	9.460	10.83	0.969
IT	31	16.13	9.420	1.810	41.70	13.74
SVK	8	11.25	4.455	1.370	33.77	13.50

PT	8	12.81	7.018	3.550	32.64	12.10
ES	15	18.55	10.55	2.370	59.18	17.05
HU	4	24.20	26.73	8.940	34.42	11.71
AU	7	11.25	7.080	3.350	33.24	10.34
SA	2	5.965	5.965	2.720	9.210	4.589
BG	5	28.80	32.19	6.370	41.12	13.13
MD	3	10.86	4.955	1.820	31.70	14.13
CN	2	4.355	4.355	4.050	4.660	0.432
AR	1	9.700	9.700	9.700	9.700	-
AT	3	20.71	10.10	10.09	41.94	18.39
AM	1	2.750	2.750	2.750	2.750	-
DE	5	22.30	32.37	5.680	33.85	15.09
GR	2	17.81	17.81	4.670	30.94	18.58
NZ	1	9.470	9.470	9.470	9.470	-
UA	1	34.77	34.77	34.77	34.77	-
CZ	1	6.920	6.920	6.920	6.920	-
UK	1	4.710	4.710	4.710	4.710	14.34

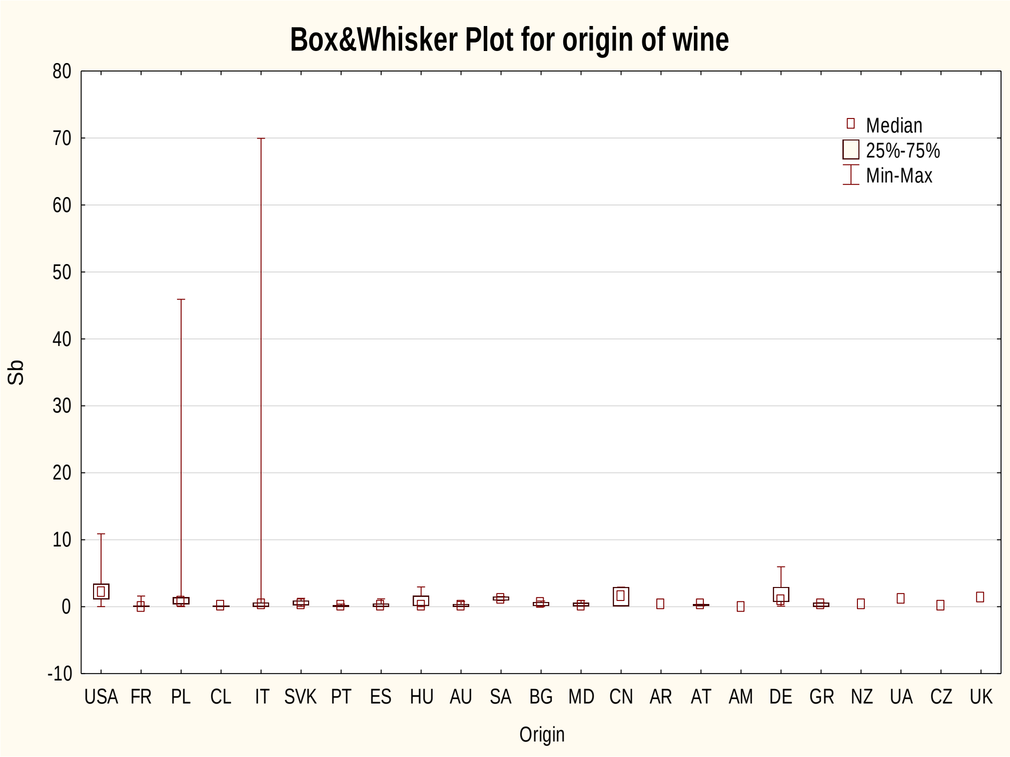

Figure S19. Box&Whisker Plot for content of Sb for obtained results of 180 samples of wine with division according to country of origin [µg/L].

Table S19. Basic statistics of Sb for all wine samples with division according to country of origin (n = 180) [µg/L].

Origin	N	Mean	Median	Min	Max	Std. Dev.
USA	18	2.752	2.147	<LOD	10.88	2.426
FR	16	0.187	<LOD	<LOD	1.584	0.411
PL	42	2.181	0.815	<LOD	45.91	7.025

CL	2	0.059	0.059	<LOD	0.109	0.070
IT	31	2.603	0.275	<LOD	69.95	12.51
SVK	8	0.561	0.451	0.051	1.246	0.423
PT	8	0.146	0.113	<LOD	0.389	0.150
ES	15	0.311	0.202	<LOD	1.166	0.365
HU	4	0.862	0.238	<LOD	2.962	1.404
AU	7	0.223	0.065	<LOD	0.767	0.287
SA	2	1.221	1.221	0.935	1.507	0.405
BG	5	0.432	0.512	<LOD	0.848	0.365
MD	3	0.326	0.153	0.075	0.925	0.403
CN	2	1.510	1.510	0.099	2.921	1.996
AR	1	0.266	0.266	0.266	0.266	-
AT	3	0.262	0.308	0.124	0.354	0.122
AM	1	0.019	0.019	0.019	0.019	-
DE	5	2.124	0.925	0.075	5.966	2.399
GR	2	0.297	0.297	<LOD	0.584	0.406
NZ	1	0.302	0.302	0.302	0.302	-
UA	1	1.217	1.217	1.217	1.217	-
CZ	1	0.084	0.084	0.084	0.084	-
UK	1	1.377	1.377	1.377	1.377	-

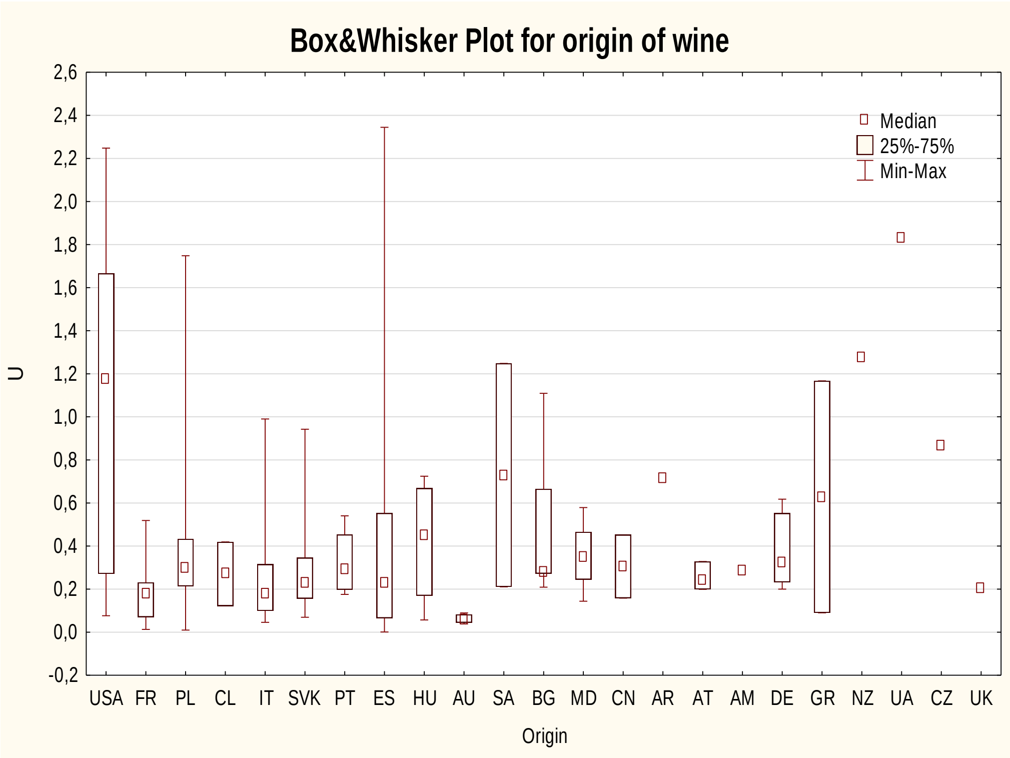

Figure S20. Box&Whisker Plot for content of U for obtained results of 180 samples of wine with division according to country of origin [μg/L].

Table S20. Basic statistics of U for all wine samples with division according to country of origin (n = 180) [μg/L].

Origin	N	Mean	Median	Min	Max	Std. Dev.

USA	18	1.028	1.175	0.077	2.248	0.721
FR	16	0.181	0.181	0.013	0.519	0.131
PL	42	0.367	0.298	<LOD	1.748	0.300
CL	2	0.270	0.270	0.122	0.419	0.210
IT	31	0.239	0.178	0.046	0.990	0.190
SVK	8	0.309	0.229	0.070	0.943	0.276
PT	8	0.325	0.290	0.176	0.540	0.149
ES	15	0.427	0.226	<LOD	2.345	0.582
HU	4	0.419	0.447	0.057	0.725	0.306
AU	7	0.061	0.057	0.038	0.090	0.019
SA	2	0.729	0.729	0.210	1.248	0.734
BG	5	0.507	0.280	0.209	1.109	0.382
MD	3	0.355	0.349	0.144	0.579	0.178
CN	2	0.305	0.305	0.158	0.452	0.208
AR	1	0.715	0.715	0.715	0.715	-
AT	3	0.256	0.241	0.199	0.328	0.066
AM	1	0.283	0.283	0.283	0.283	-
DE	5	0.386	0.327	0.200	0.618	0.189
GR	2	0.629	0.629	0.090	1.167	0.762
NZ	1	1.275	1.275	1.275	1.275	-
UA	1	1.829	1.829	1.829	1.829	-
CZ	1	0.869	0.869	0.869	0.869	-
UK	1	0.204	0.205	0.205	0.205	-

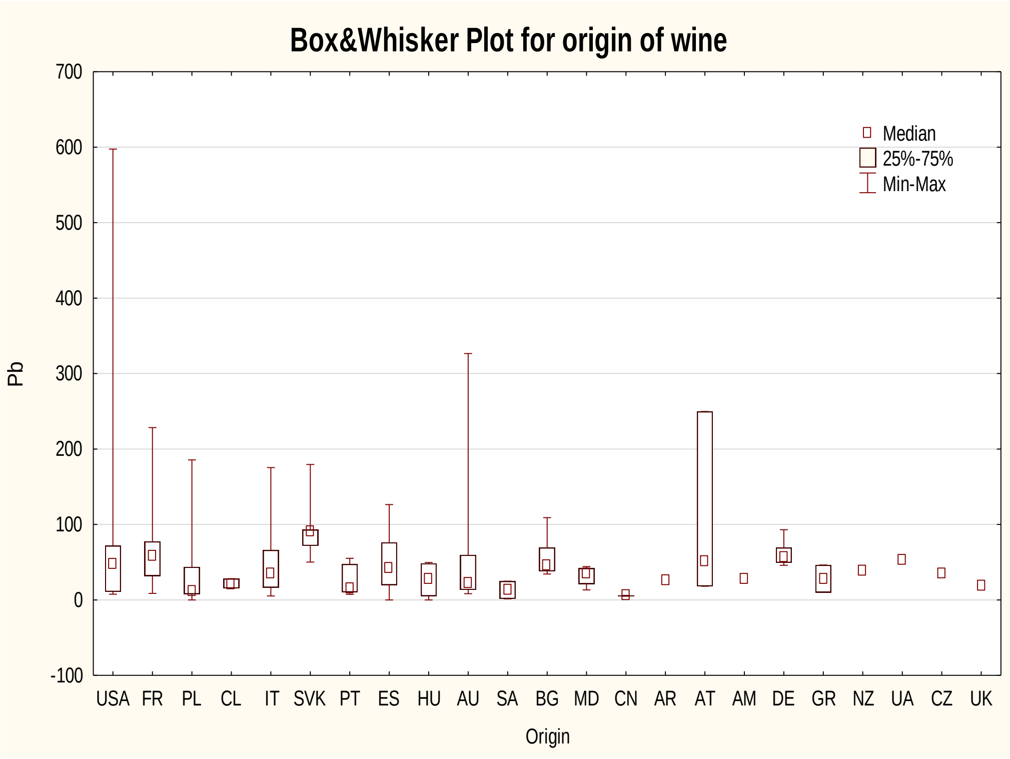

Figure S21. Box&Whisker Plot for content of Pb for obtained results of 180 samples of wine with division according to country of origin [μg/L].

Table S21. Basic statistics of Pb for all wine samples with division according to country of origin (n = 180) [µg/L].

Origin	N	Mean	Median	Min	Max	Std. Dev.
USA	18	88.53	48.02	7.512	597.6	141.0
FR	16	64.74	59.23	8.730	228.5	51.77
PL	42	31.77	12.22	<LOD	185.6	42.41
CL	2	21.80	21.80	15.40	28.19	9.044
IT	31	45.26	35.09	5.310	175.3	37.37
SVK	8	92.83	91.59	50.11	179.6	38.43
PT	8	26.09	15.39	7.400	55.16	20.13
ES	15	49.76	42.59	<LOD	126.4	38.85
HU	4	26.81	28.79	<LOD	49.66	25.40
AU	7	69.41	22.66	8.300	326.5	114.6
SA	2	13.23	13.23	1.520	24.94	16.56
BG	5	59.32	45.81	34.38	109.1	30.95
MD	3	31.50	34.49	13.25	44.14	13.81
CN	2	5.513	5.513	4.980	6.047	0.754
AR	1	25.78	25.78	25.78	25.78	-
AT	3	106.3	50.93	18.13	249.7	125.3
AM	1	28.06	28.06	28.06	28.06	-
DE	5	62.96	57.17	45.87	93.01	19.07
GR	2	28.21	28.21	10.06	46.37	25.68
NZ	1	39.26	39.26	39.26	39.26	-
UA	1	53.09	53.09	53.09	53.09	-
CZ	1	34.64	34.64	34.64	34.64	-
UK	1	18.65	18.65	18.65	18.65	-

Abbreviations:

USA – United States of America

FR – France

PL – Poland

CL – Chile

IT – Italy

SVK – Slovakia

PT – Portugal

ES – Spain

HU – Hungary

AU – Australia

SA – South Africa

BG – Bulgaria

MD – Moldova

CN – China

AR – Argentina

AT – Austria

AM – Armrnia

DE – Germany

GR – Grecce

NZ – New Zealand

UA – Ukraine

CZ – Czech Republic

UK – United Kingdom

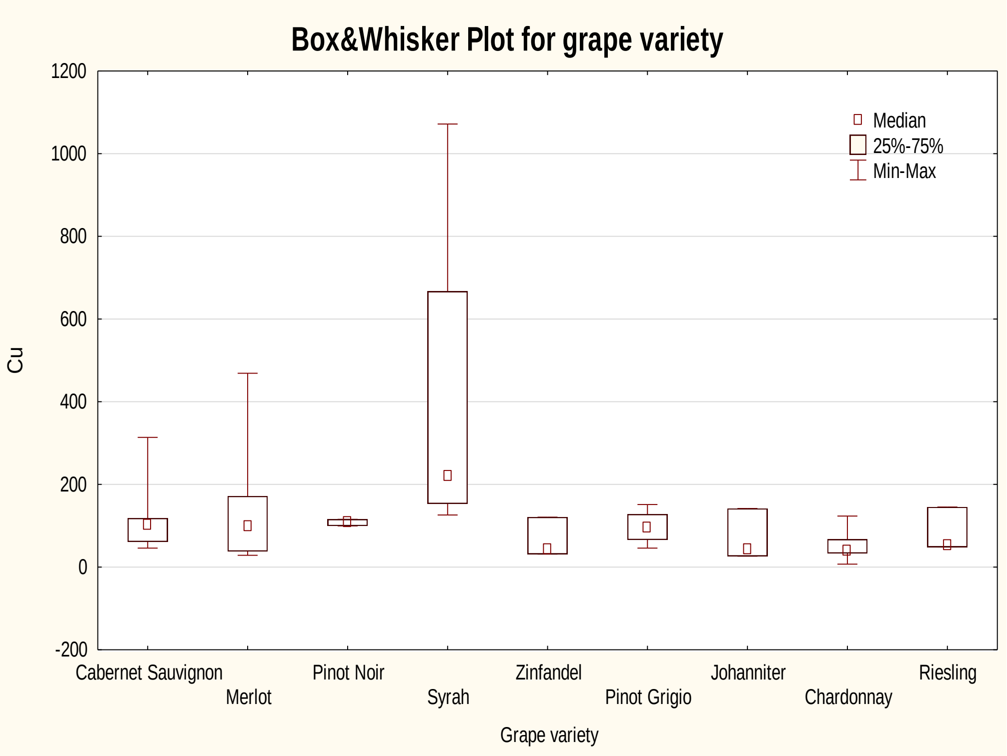

Figure S22. Box&Whisker Plot for content of Cu for obtained results of 38 samples of wine with division according to grape variety [μg/L].

Table S22. The basic statistic information of Cu content in five grape variety (n=38) [μg/L].

Grape variety	N	Mean	Median	Min	Max	Grape variety	N	Mean	Median	Min	Max
Cabernet Sauvignon	5	128.8	104.1	46.05	313.9	Pinot Grigio	4	96.92	95.19	45.92	151.4

Merlot	5	161.7	100.8	28.49	469.0	Johanniter	3	70.77	44.09	26.55	141.7
Pinot Noir	2	107.7	107.7	99.77	115.5	Chardonnay	9	54.68	39.95	7.180	123.3
Syrah	4	410.2	221.3	125.9	1072	Riesling	3	82.21	52.69	48.60	145.3
Zinfandel	3	64.75	41.95	31.32	120.9						

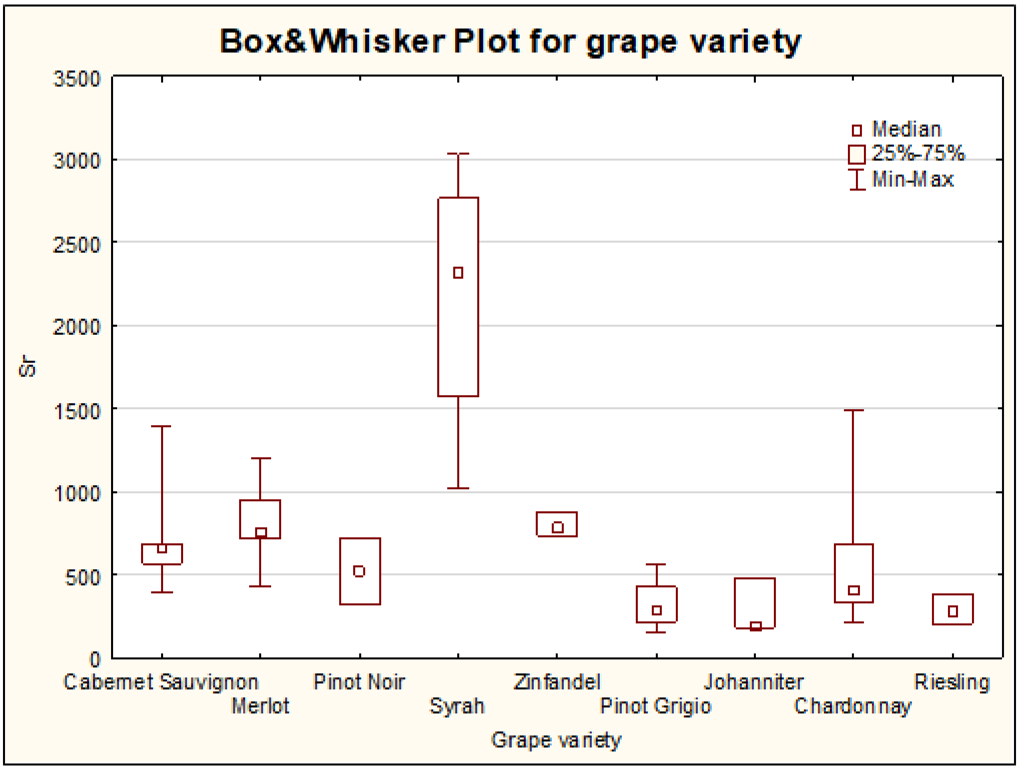

Figure S23. Box&Whisker Plot for content of Sr for obtained results of 38 samples of wine with division according to grape variety [μg/L].

Table S23. The basic statistic information of Sr content in five grape variety (n=38) [μg/L].

Grape variety	N	Mean	Median	Min	Max	Grape variety	N	Mean	Median	Min	Max
Cabernet Sauvignon	5	743.7	664.9	397.0	1402	Pinot Grigio	4	324.6	289.7	159.1	559.9
Merlot	5	814.6	762.8	433.8	1200	Johanniter	3	288.8	194.9	185.7	485.7
Pinot Noir	2	524.9	524.9	322.8	726.9	Chardonnay	9	613.8	404.0	214.8	1494
Syrah	4	2170	2311	1025	3033	Riesling	3	292.1	282.1	209.0	385.3
Zinfandel	3	803.1	786.8	739.6	882.8						

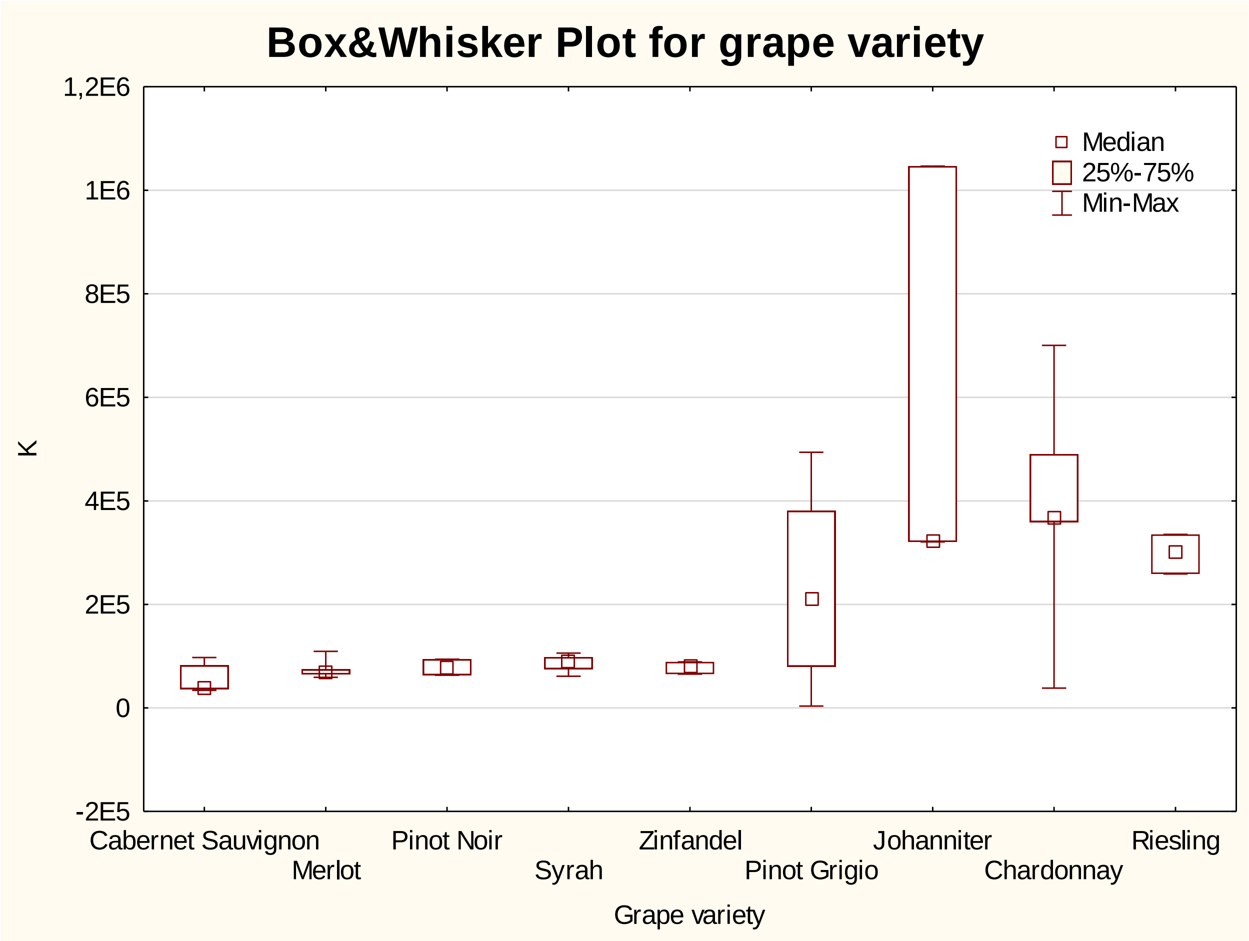

Figure S24. Box&Whisker Plot for content of K for obtained results of 38 samples of wine with division according to grape variety [μg/L].

Table S24. The basic statistic information of K content in five grape variety (n=38) [μg/L].

Grape variety	N	Mean	Median	Min	Max	Grape variety	N	Mean	Median	Min	Max
Cabernet Sauvignon	5	57488	37448	34082	97261	Pinot Grigio	4	230314	211920	3562	493856
Merlot	5	75306	68676	59101	109371	Johanniter	3	562940	321233	320800	1046788
Pinot Noir	2	78646	78646	63249	94042	Chardonnay	9	393750	366750	38250	700228
Syrah	4	86492	89514	61154	105787	Riesling	3	298287	300724	258826	335310
Zinfandel	3	78259	80496	65331	88950						

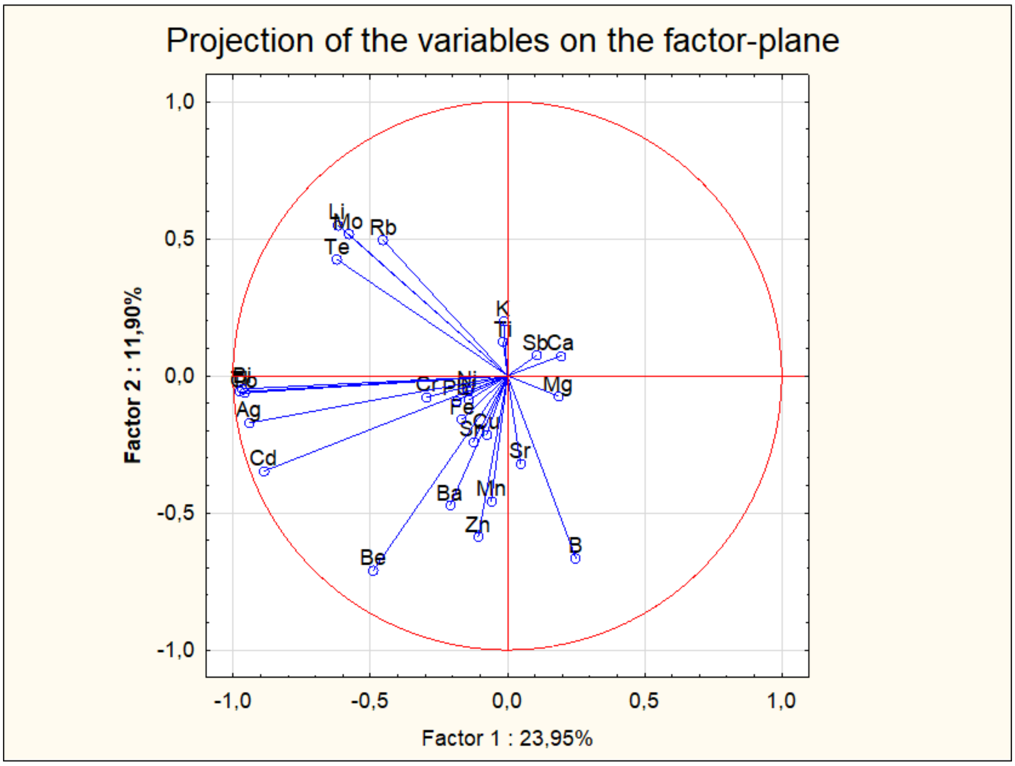

Figure S25. Projection of the variables on the factor-plane in 180 samples investigated in this study for the whole data set [μg/L].

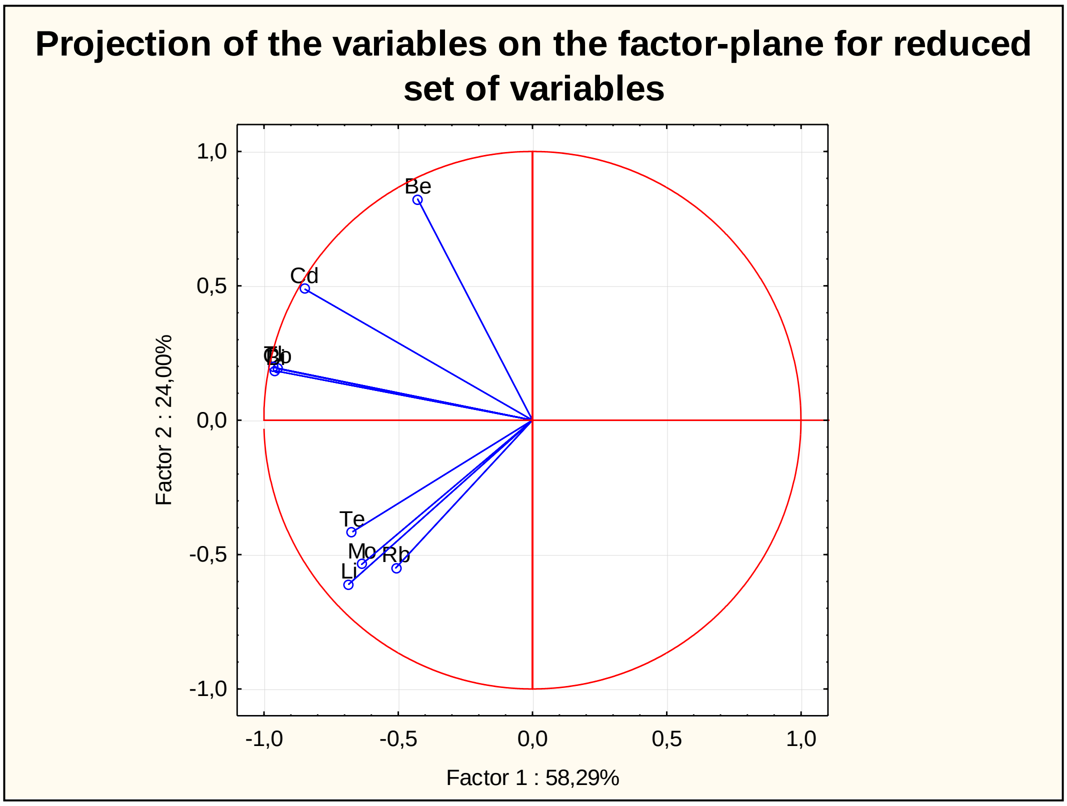

Figure S26. Projection of the variables on the factor-plane in 180 samples investigated in this study for the reduced data set [μg/L].

Table S25. The characterization of the wine samples

Sample Code	Type	Price [€/0.75L]	Alcohol content [%]	Grape variety	Origin
1R	Red – Semi Sweet	5.5	11	Concord	USA - Pennsylvania
2R	Red - Dry	8.5	0	Cabernet Sauvignon	France - Languedoc
3R	Red - Sweet	4	14	Multifruit	Poland - Dobron
4R	Red - Dry	6	13	Merlot	Chile
5R	Red – Semi Dry	6	12.5	Ruby Cabernet	USA - California
6R	Red - Dry	17	13.5	Pinot Noir	France - Languedoc
7R	Red - Dry	16	14	Sangiovese, Sagrantino, Merlot	Italy - Umbria
8R	Red – Semi Sweet	6.5	11	Multifruit	Slovakia
9R	Red - Dry	6.5	12	Alibernet	Slovakia - Moravia
10R	Red - Dry	6.5	13.5	Castelao, Tinta Roriz	Portugal - Lisbon
11R	Red - Dry	6.5	12.5	Cabernet Sauvignon	Slovakia - Dolné Ohňavy
12R	Red - Dry	6	12.5	Tempranillo	Spain - Castilla
13R	Red - Dry	14	13	Pinot Noir	Hungary - Matra
14R	Red - Dry	6	12	Primitivo	Italy - Puglia
15R	Red - Dry	10.5	14	Cabernet Franc	France - Languedoc
16R	Red - Dry	6	13.5	Tempranillo	Spain - Carinera
17R	Red - Sweet	6	9	Multifruit	USA - California

18R	Red - Dry	14	15	Zinfandel, Petite Sirah, Merlot, Malbec	USA - California
19R	Red - Dry	8	13.5	Syrah	Australia - South - East
20R	Red - Dry	19.5	14	Cabernet Syrah	France - Languedoc
21R	Red - Dry	17.5	13	Cabernet Sauvignon	Chile – Maipo Valley
22R	Red - Dry	13	14	Syrah	Australia - South - East
23R	Red - Dry	7	13.5	Multifruit	Slovakia - Dolné Ohňavy
24R	Red - Dry	25.5	15	Multifruit	Spain - Priorat
25R	Red - Dry	17.5	13	Susumaniello	Italy - Puglia
26R	Red - Dry	9	13.5	Merlot, Cabernet Franc	Italy - Tuscany
27R	Red - Dry	20	14	Syrah	Australia – Barossa Valley
28R	Red - Dry	17.5	13	Nerello Mascalese, Nerello Cappuccio	Italy - Sicilia
29R	Red - Dry	6	13	Merlot, Cabernet Sauvignon, Cabernet Franc	France - Bordeaux
30R	Red - Dry	6	13	Multifruit	USA - California
31R	Red - Sweet	5	11	Multifruit	Poland -Dobron
32R	Red – Semi Sweet	7	10.5	Alicante Bouschet, Tinta Roriz, Touriga Nacional	Portugal - Lisbon
33R	Red - Sweet	5	11	Multifruit	Poland -Dobron
34R	Red - Sweet	5	10	Multifruit	USA - Pennsylvania
35R	Red - Dry	16	12	Merlot	Italy - Veneto
36R	Red - Sweet	5	11	Multifruit	Poland -Dobron

37R	Red - Sweet	2.5	14	Multifruit	Poland - Torun
38R	Red - Sweet	7	20	Multifruit	Portugal - Porto
39R	Red – Semi Sweet	5	13	Primitivo	Italy - Salento
40R	Red - Dry	7	-	-	France
41R	Red – Semi Sweet	7	11	Le Carillon	France -Loire Valley
42R	Red - Sweet	45	17	Pedro Ximenez	Spain - Andalusia
43R	Red - Dry	8.5	13	Dolcetto	Italy - Monferrato
44R	Red - Dry	12	13.5	Montepulciano	Italy - Abruzzo
45R	Red - Dry	24.5	14	Merlot	Italy - Tuscany
46R	Red - Dry	14.5	13	Listán Negro	Spain - Tenerife
47R	Red – Semi Dry	8.5	14	Cabernet Sauvignon, Merlot	Australia - South - East
48R	Red - Dry	7.5	13.5	Grenache, Syrah	France - Languedoc
49R	Red - Dry	12.5	13.5	Barbera	Italy - Tuscany
50R	Red - Dry	14	15.9	Zinfandel	USA – Dry Creek Valley
51R	Red - Sweet	14.5	9	Terrano	Italy - Romagna
52R	Red – Semi Sweet	3.5	13	Merlot	South Africa – Western Cape
53R	Red – Semi Dry	6	13	Mataro, Syrah	Australia - South - East
54R	Red – Semi Dry	4.5	14	Syrah	Australia - South - East
55R	Red – Semi Dry	7	13	Cabernet Sauvignon, Syrah	France - Languedoc
56R	Red – Semi Dry	3.5	13.5	Merlot	USA - California

57R	Red - Dry	4.5	12	Multifruit	Bulgaria - Eger
58R	Red - Sweet	-	-	Multifruit	Poland - Lodz
59R	Red - Sweet	-	-	Multifruit	Poland - Skierniewice
60R	Red – Semi Dry	4.5	12	Cabernet Sauvignon	Bulgaria - Valley of Roses
61R	Red - Dry	4.5	13	Cabernet Sauvignon, Merlot	Moldova - Ethulia
62R	Red – Semi Sweet	7	12.5	Alicante Bouschet, Tinta Roriz, Touriga Nacional	Portugal - Lisbon
63R	Red – Semi Dry	11.5	13.5	Negroamaro	Italy - Apulia
64R	Red - Sweet	3.5	11.5	Tempranillo, Shiraz	Spain
65R	Red – Semi Sweet	4.5	11.5	Cabernet Sauvignon	Moldova - Ethulia
66R	Red - Sweet	4.5	14.5	Multifruit	Poland
67R	Red - Dry	17.5	13.5	Garnacha, Tempranillo	Spain - La Rioja
68R	Red - Dry	13	13.5	Barbera	Italy - Piedmont
69R	Red - Sweet	12	19.5	Multifruit	Portugal - Lisbon
70R	Red - Sweet	3.5	12	Multifruit	Poland
71R	Red - Sweet	2.5	13	Multifruit	Poland
72R	Red - Sweet	-	-	Multifruit	Poland - Legionowo
73R	Red - Dry	3.5	12	Carmenere	Chile
74R	Red - Dry	3.5	12.5	Sangiovese, Canaiolo	Italy - Tuscany
75R	Red - Sweet	4.5	13.5	Multifruit	Poland

76R	Red - Sweet	6	9	Multifruit	USA - California
77R	Red – Semi Dry	4.5	12	Bobal	Spain - Requena
78R	Red – Semi Sweet	3.5	5.5	Multifruit	Poland
79R	Red - Sweet	4.5	13.5	Multifruit	Poland
1W	White - Dry	14	12.5	Pinot Grigio	Italy - Veneto
2W	White – Sweet	46.5	7.5	Johanniter	Poland - Baniewice
3W	White - Dry	23.5	11.5	Garnacha Blanco	Spain - Priorat
4W	White - Dry	9.5	11.5	Chardonnay	France - Gascogne
5W	White – Sweet	18	16	Malvasia	Italy - Sicilia
6W	White - Dry	14	11.5	Tempranillo, Garnacha	Spain - Rioja
7W	White - Dry	6.5	12	Muller Thurgau	Italy - Adyga
8W	White – Semi Dry	6.5	13	Trebbiano	Slovakia - Dolné Ohňavy
9W	White - Dry	6.5	12	Morawski Muscat	Slovakia - Moravia
10W	White - Dry	6	11.5	Sauvignon Blanc, Chardonnay, Verdejo	Spain - Castilla
11W	White - Dry	10	12.5	Sauvignon Blanc	Chile - Valparaiso
12W	White - Dry	11.5	12.5	Chardonnay	Argentina - Mendoza
13W	White - Dry	16	11.5	Gruner Veltliner	Austria - Wachau
14W	White - Dry	12	14	Pecorino	Italy - Offida
15W	White - Dry	8	11.5	Sauvignon Blanc, Ugni Blanc, Colombard	France - Gascogne
16W	White – Semi Dry	7	12	Chardonnay	Bulgaria - Stara Zagora

17W	White – Sweet	6	10	Multifruit	USA - Pennsylvania
18W	White - Dry	17.5	12	Kangun	Armenia - Aragatsotn
19W	White - Dry	14	12.5	Chardonnay	Portugal - Tejo
20W	White - Dry	7	12	Muller Thurgau	Slovakia - Dolné Ohňavy
21W	White – Semi Dry	7	0	Multifruit	Germany - Moguncja
22W	White – Semi Dry	9.5	12.5	Irsai Oliver	Slovakia
23W	White – Semi Dry	7	11	Multifruit	Italy - Veneto
24W	White – Semi Dry	11.5	12.5	Merseguera, Moscatel	Spain - Alicante
25W	White - Dry	14	13	Malvasia	Italy - Emilia
26W	White – Semi Sweet	11.5	10	Riesling	Germany - Mosel
27W	White - Dry	10	12.5	Johanniter	Poland - Zbyczyna
28W	White - Dry	6	12	Pinot Grigio	Italy – Delle Venezie
29W	White – Semi Sweet	4.5	10	Multifruit	USA - Pennsylvania
30W	White – Semi Sweet	3.5	11.5	Muscat Ottonel	Hungary - Kunsag
31W	White - Dry	47	12	Pinot Grigio	Italy - Veneto
32W	White – Sweet	2.5	-	Chardonnay	Moldova - Onesti
33W	White – Sweet	4.5	10	Multifruit	Poland - Dobron
34W	White – Semi Dry	6	11.5	Chardonnay	Bulgaria
35W	White – Sweet	9.5	7.5	Multifruit	Italy - Asti
36W	White – Sweet	2.5	14	Multifruit	Poland - Torun

37W	White - Dry	7.5	13	Rolle	France - Provence
38W	White - Dry	21	12	Furmint, Chardonnay	Hungary – Nagy Somló
39W	White - Dry	14	11	Xarel-lo, Parellada, Macabeu	Spain - Cava
40W	White - Dry	24.5	13	Roditis	Greece - Pella
41W	White - Dry	10	13.5	Garganega	Italy - Soave
42W	White - Dry	11.5	0	Riesling	Germany - Rhringau
43W	White - Dry	11.5	12.5	Furmint, Hárslevelű, Sárgamuskotály	Hungary - Tokaj
44W	White – Semi Dry	12.5	12.5	Gewurztraminer	New Zealand - Nelson
45W	White - Dry	17.5	11.5	Johanniter	Poland - Sandomierz
46W	White - Dry	16	12	Riesling	Poland - Lower Silesia
47W	White - Dry	8.5	13	Chardonnay	France - Languedoc
48W	White – Semi Sweet	8	15	Multifruit	Italy - Turin
49W	White - Dry	6	12.5	Chardonnay	Australia - South - East
50W	White – Sweet	7	13	-	Poland – Nowy Sacz
51W	White – Semi Sweet	6	9.5	-	Germany - Nahe
52W	White – Semi Sweet	4.5	12	Cabernet Sauvignon	Bulgaria - Valley of Roses
53W	White – Semi Dry	4.5	13	Sauvignon Blanc	Moldavia
54W	White - Dry	6	13	Chenin Blanc	South Africa – Western Cape
55W	White - Dry	8	10.5	Colombard, Gros Manseng, Sauvignon Blanc, Ugni Blanc	France - Gascogne

56W	White - Dry	8	12.5	Insolia, Cataratto, Grillo	Italy - Sicilia
57W	White - Dry	8	12.5	Gewurztraminer	Spain - Aragonia
58W	White – Dry	8	12	Loureiro, Arinto	Portugal - Minho
59W	White – Semi Dry	6	10.5	Chardonnay	Ukraine
60W	White - Sweet	4.5	14	Multifruit	Poland
61W	White - Sweet	3.5	14.5	Multifruit	Poland
62W	White - Sweet	4.5	14	Multifruit	Poland
63W	White – Semi Sweet	4.5	11	Multifruit	Greece - Crete
64W	White – Semi Sweet	3.5	10	Multifruit	Poland
65W	White – Semi Dry	3.5	10	Multifruit	Poland
66W	White - Sweet	3.5	5.5	Multifruit	Poland
67W	White – Semi Dry	2.5	7.5	Multifruit	Italy - Arduini
68W	White - Dry	4.5	12	Pinot Grigio	Italy - Veneto
69W	White – Semi Sweet	2.5	9	Multifruit	Poland
70W	White - Sweet	2.5	10	Multifruit	Poland
71W	White - Sweet	3.5	10.5	Multifruit	Poland
72W	White - Dry	7	14	Chardonnay, Arinto	Portugal - Tejo
73W	White - Sweet	6	15	Multifruit	Poland
74W	White - Sweet	2.5	15	Multifruit	Poland
75W	White - Sweet	2.5	10	Multifruit	Poland

1Ro	Rosé - Dry	10	12.5	Grenache, Syrah	France - Languedoc
2Ro	Rosé - Sweet	6	10	Multifruit	USA - California
3Ro	Rosé - Dry	11.5	11	Grauburgunder, Neuburger, Pinot Blanc	Austria – Burgenland
4Ro	Rosé – Semi Sweet	7	10	Multifruit	USA - California
5Ro	Rosé - Sweet	4.5	10	Multifruit	Poland - Dobron
6Ro	Rosé - Sweet	4.5	10	Multifruit	Poland - Dobron
7Ro	Rosé – Semi Sweet	4.5	10	Zinfandel	USA - Pennsylvania
8Ro	Rosé – Semi Dry	7	-	-	France
9Ro	Rosé - Dry	7	11.5	Svatovavrinecke	Czech Republic - Moravia
10Ro	Rosé – Semi Dry	7	10	Multifruit	USA - California
11Ro	Rosé – Semi Dry	3.5	10.5	Zinfandel	USA - California
12Ro	Rosé - Sweet	3.5	9	Multifruit	UK
13Ro	Rosé - Sweet	12	7.5	Muscato	Italy - Veneto
14Ro	Rosé - Dry	12	11	Grauburgunder, Neuburger, Pinot Blanc	Austria – Burgenland
15Ro	Rosé - Sweet	4.5	10	Multifruit	Poland - Dobron
16Ro	Rosé - Dry	10	11.5	-	Germany - Kirrweiler
17Ro	Rosé - Dry	18	11.5	Pinot Noir, Grenache, Cava Rimarts	Spain - Catalonia
18Ro	Rosé – Semi Sweet	3.5	10	Multifruit	Poland
19Ro	Rosé - Sweet	2	12	Multifruit	Poland
20Ro	Rosé - Sweet	4.5	10	Multifruit	Poland - Dobron

21Ro	Rosé - Sweet	6	10	Multifruit	USA - California
22Ro	Rosé - Sweet	6	10	Multifruit	USA - California
23Ro	Rosé - Sweet	4.5	9.5	Multifruit	Poland
24Ro	Rosé - Sweet	7	10	Multifruit	Poland - Dobron
25Ro	Rosé – Semi Sweet	7	11	Multifruit	Italy
26Ro	Rosé – Sweet	3.5	5.5	Multifruit	Poland
